# The genetic legacy of the first successful reintroduction of a mammal to Britain: Founder events and attempted genetic rescue in Scotland's beaver population

**DOI:** 10.1111/eva.13629

**Published:** 2023-12-28

**Authors:** Helen R. Taylor, Jean‐Marc Costanzi, Kara L. Dicks, Helen V. Senn, Sarah Robinson, Gill Dowse, Alex D. Ball

**Affiliations:** ^1^ Field Conservation Royal Zoological Society of Scotland Edinburgh UK; ^2^ WildGenes Laboratory Royal Zoological Society of Scotland Edinburgh UK; ^3^ Microbiology and Infection Control Akershus University Hospital Oslo Norway; ^4^ Scottish Wildlife Trust Edinburgh UK

**Keywords:** conservation, genetic rescue, mammal, reintroduction, relatedness, translocation

## Abstract

Conservation translocations often inherently involve a risk of genetic diversity loss, and thus loss of adaptive potential, but this risk is rarely quantified or monitored through time. The reintroduction of beavers to Scotland, via the Scottish Beaver Trial in Knapdale, is an example of a translocation that took place in the absence of genetic data for the founder individuals and resulted in a small and suspected to be genetically depauperate population. In this study we use a high‐density SNP panel to assess the genetic impact of that initial translocation and the effect of subsequent reinforcement translocations using animals from a different genetic source to the original founders. We demonstrate that the initial translocation did, indeed, lead to low genetic diversity (*H*_o_ = 0.052) and high mean kinship (KING‐robust = 0.159) in the Knapdale population compared to other beaver populations. We also show that the reinforcement translocations have succeeded in increasing genetic diversity (*H*_o_ = 0.196) and reducing kinship (KING robust = 0.028) in Knapdale. As yet, there is no evidence of admixture between the two genetic lineages that are now present in Knapdale and such admixture is necessary to realise the full genetic benefits of the reinforcement and for genetic reinforcement and then rescue to occur; future genetic monitoring will be required to assess whether this has happened. We note that, should admixture occur, the Knapdale population will harbour combinations of genetic diversity not currently seen elsewhere in Eurasian beavers, posing important considerations for the future management of this population. We consider our results in the wider context of beaver conservation throughout Scotland and the rest of Britain, and advocate for more proactive genetic sampling of all founders to allow the full integration of genetic data into translocation planning in general.

## 1 INTRODUCTION

In the current biodiversity crisis, species reintroductions are a popular and necessary conservation strategy, but are also high risk and prone to failure (Bellis et al., 2019; Bubac et al., 2019; Godefroid et al., 2011). Reasons for failure vary (Berger‐Tal et al., 2020), but genetic factors such as loss of diversity (and thus adaptive potential) linked to low founder numbers are frequently cited as an issue for reintroduced populations, especially in the longer term (Bellis et al., 2019; Biebach & Keller, 2010; Murphy et al., 2021; Taylor, Colbourne, et al., 2017). Such populations are often necessarily founded with a relatively small number of individuals (Tracy & Jamieson, 2011). Source populations for founders are sometimes, themselves, relatively small, and thus cannot support more than a small number of individuals being removed for a reintroduction attempt (Easton et al., 2020). Risks of unanticipated consequences for species absent from a landscape for generations and a lack of resources can also necessitate a small‐scale trial approach to reintroduction efforts (Kemp et al., 2015).

Genetic monitoring should be an important component of reintroduction programmes but is often missing. Reintroduced populations with small founder numbers are unlikely to capture the genetic diversity present in the source population (Weeks et al., 2015; Weiser et al., 2013). Such reintroduced populations are also more vulnerable to inbreeding, and reproductive skew within the population due to stochastic events (Weeks et al., 2015). These issues could result in a need for reinforcement translocations at a later stage to effect genetic rescue (i.e., population growth following improved genetic diversity; Tallmon et al., 2004), but assessing genetic diversity to aid decisions around genetic management and evaluate the efficacy of any management actions requires genetic data. These data are frequently lacking from reintroduction programmes due to issues around funding, the time required to obtain genetic data, lack of long‐term sample collection commitment, and the general disconnect between conservation genetic research and conservation management (Taylor, Dussex, et al., 2017). In addition, some reintroductions are unauthorised, and thus not necessarily planned with genetic management (or long‐term management of any kind) in mind (e.g., Crowley et al., 2017; Pucci et al., 2021). Reintroduction programmes that bridge this gap and manage to collect genetic data from source populations, at founding, and over the course of a reintroduction effort can be a vital part of assessing the success of the reintroduction and helping inform future management needs, including the necessity of genetic rescue, for the reintroduced population.

**FIGURE 1:**
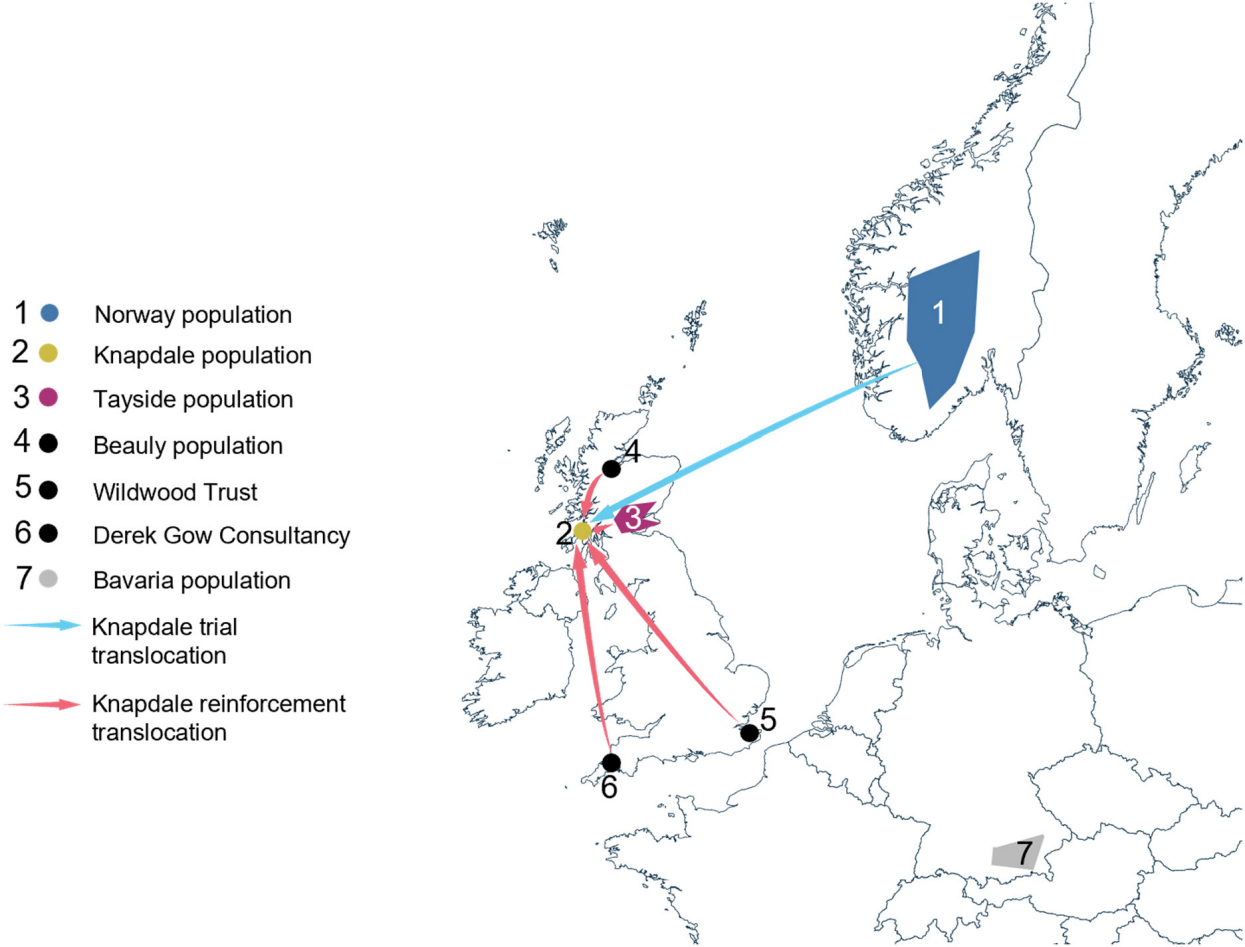
Locations of beaver populations in this study and translocations between populations. Note that, as the route of Bavarian stock beavers into Britain is not currently fully documented, we have not attempted to hypothesise this route here. Only known, documented translocation routes are shown.

The reintroduction of the Eurasian beaver (*Castor fiber*) to Scotland represents both a landmark reintroduction effort and an example of genetic data collection throughout a reintroduction programme. The first mammal to be successfully reintroduced to the UK, beavers had been absent from Britain for over 400 years, having been extirpated via a combination of hunting and land‐use change (Kitchener & Conroy, 1997). Reintroducing beavers to Scotland was controversial; they are ecosystem engineers that can bring wide ranging biodiversity benefits (Stringer & Gaywood, 2016), but can also flood out agricultural land and damage property (Campbell‐Palmer et al., 2015). Thus, the initial licensed reintroduction of beavers to Scotland involved a small‐scale trial, bringing a total of 16 beavers to Knapdale forest on the West Coast of Scotland from Telemark in Norway, as part of the Scottish Beaver Trial (Campbell‐Palmer & Jones, 2014) (Figure 1).

Although genetic material was collected throughout the Scottish Beaver Trial, a full genetic study of Eurasian beavers was not conducted until after the Trial had finished, due in part to low microsatellite polymorphism for beavers and a lack of other tools available at the time (H. Senn, personal observations). This analysis suggested Norway was a suboptimal source population for a beaver reintroduction (Senn et al., 2014). Norway was selected as the sole source population for the Knapdale Trial due to morphological similarities in beaver skulls found in Britain and those in Norway (Kitchener & Lynch, 2000). However, beavers in Norway were, themselves, hunted to near extinction by the 1880s (Collett, 1897); the resulting population bottleneck has led to relatively low genetic diversity in Norwegian beavers compared to those in other locations across Europe (Senn et al., 2014). Moreover, at the end of Scottish Beaver Trial in 2014, just nine beavers were known to be present at the trial site, increasing the risks of further loss of genetic diversity and the probability of inbreeding in the reintroduced Knapdale population.

While the licensed Scottish Beaver Trial was taking place, separate, unauthorised releases of beavers occurred in multiple locations across Britain, including the River Tay catchment and the River Beauly in the east of Scotland, and on the River Otter in the south‐west of England. The River Tay population in particular grew rapidly, from a small founder group of about three families in around 2006, to an estimated 945 individuals in 2021 (Campbell‐Palmer et al., 2021). Unlike Knapdale, the vast majority of the land around the River Tay is prime agricultural land, and flooding and structural damage by beavers in the Tayside area created human wildlife conflict between landowners and beavers (Hamilton & Moran, 2015). This conflict resulted in unregulated culling and then, once beavers in Scotland received European Protected Species status in 2019, licensed lethal control of beavers in Tayside. Genetic analyses of a limited data set of animals (*n* = 43) suggested that Britain's illegally released beavers were of Bavarian origin with a very small number (represented by just one sample) of animals with DNA that corresponds to reference samples from river systems that span Lithuania, Poland, Belarus, and Russia (Campbell‐Palmer et al., 2020).

Bavarian beavers harbour more and different genetic diversity to Norwegian beavers at both mitochondrial and nuclear loci because they are, themselves, a result of a reintroduction involving individuals from a mixture of French, Norwegian, and Eastern European populations (Nolet & Rosell, 1998; Senn et al., 2014). It was thus hypothesised that translocating beavers of Bavarian origin to Knapdale could improve the genetic diversity in the Knapdale population, potentially leading to genetic rescue. While beavers have spread out of Tayside and into the Forth catchment and other areas (Campbell‐Palmer et al., 2021), it is unlikely that they would migrate into Knapdale naturally as Knapdale was specifically selected as a “closed” site, bordered to the North by the Crinan canal and by the sea on all other sides (Campbell‐Palmer & Jones, 2014). Human‐mediated movement is the only effective way of moving beavers into Knapdale. Additionally, if some animals could be sourced from Tayside for translocation, this strategy also had the potential to alleviate some of the human wildlife conflict occurring in that area.

The Scottish Beavers Reinforcement project was set up to conduct translocations of animals of Bavarian genetic stock into Knapdale. Between 2017 and 2020, 21 beavers were translocated to Knapdale (Dowse et al., 2020). The majority of animals (*N* = 14) came from conflict sites in Tayside, with a small number (*N* = 7) from the River Beauly, Wildwood Trust in Kent, and Derek Gow Consultancy (all of which were also Bavarian beavers – referred to as reinforcement beavers for the rest of this manuscript) (Figure 1). The aim of the project was that Bavarian stock beavers would establish in Knapdale and breed, offering opportunities for admixture between the two lineages, increasing genetic diversity, reducing the risk of inbreeding, and improving the fitness of the population.

It should be noted that there were originally concerns that the Bavarian beavers were not suitable for the reinforcement effort as they represented a stock mixed from both eastern and western “evolutionary significant units” (ESUs, as postulated by Durka et al. (2005)). However, analysis of ancient samples (Horn et al., 2014) revealed that much of the contemporary genetic structure found in beaver populations across Eurasia is heavily influenced by the significant range contraction into different “refugia” that they experienced when driven to virtual extinction by the fur trade in the 19th century. As a result, genetic diversity today is considerably lower, and population differences are inflated, relative to historical baselines (see Senn et al., 2014 table 1 for a summary of the history). In general, the weight of data suggest that the risks of inbreeding depression outweigh those of outbreeding depression (Frankham, 2015; Frankham et al., 2011), but many studies fail to consider the risks of inbreeding depression and outbreeding depression simultaneously when it comes to management actions, leading to overly conservative management, with populations managed separately rather than being mixed (Liddell et al., 2021). In the case of Knapdale's beavers, at the time of the reinforcement planning, it was argued that a limited risk of outbreeding depression existed because there was already genetic evidence for mixing between the postulated ESUs, both in naturally recovered and deliberately mixed populations (Scottish Natural Heritage, 2015; Senn et al., 2014), thus the idea of a reinforcement was supported.

Genetic samples are available from all source and recipient populations for beavers in Scotland. Samples from source populations in Norway and Bavaria had already been collated for a previous study (Senn et al., 2014), the Tayside population had also been sampled previously (Campbell‐Palmer et al., 2020; McEwing et al., 2015), all original Norwegian founders and the reinforcement animals going into Knapdale were sampled for DNA (this study), and an intensive capture and sampling effort took place in Knapdale in 2019 to establish a current snapshot of genetic diversity in that population (this study). Here, we analyse these samples via a reduced‐representation sequencing protocol, double digest restriction‐site associated DNA (ddRAD), to ask:

1. What (if any) genetic diversity was lost when beavers were translocated from Norway to Knapdale, and Bavaria to Tayside?
2. What (if any) population‐level genetic diversity has been gained by translocating Bavarian‐stock beavers into Knapdale?
3. Is there any evidence of admixture between Norwegian and Bavarian‐stock beavers within the Knapdale population that could lead to genetic rescue at this point?

By establishing the genetic legacy of the various reintroductions and translocations of beavers into and around Scotland, we assess the potential need for more genetic reinforcement/rescue translocations in the future. We also suggest ways forward for the management of beavers in Scotland and beyond, while highlighting the importance of genetic data in effective reintroduction management.

## 2 METHODS

### 2.1 Sample collection

The DNA from beavers in the source populations in Norway and Bavaria was sampled as described in Senn et al. (2014). For Scottish populations, beaver DNA was collected either as part of health screening requirements or, in the case of Knapdale‐born individuals, via live trapping and sampling. In all cases involving live animals, DNA was obtained from blood samples taken from the tail of each beaver by a qualified vet. The exceptions to this were DNA samples obtained from beavers that had perished – in these cases, tissue samples were taken during autopsy by a qualified vet. We stored blood samples in EDTA and tissue samples in ethanol. We kept all samples at −20°C prior to DNA extraction. A full list of samples, origins, and genetic sequence accessions can be found in the Table S1. All beaver trapping and translocations that formed part of this study were undertaken under license from NatureScot (previously Scottish Natural Heritage).

### 2.2 Sequencing and variant calling

#### 2.2.1 DNA extractions

DNA extractions from blood and tissue were conducted using the manufacturer's protocols according to sample type using one of the following kits: QuickGene DNA whole blood kit S (FUJIFILM Wako Chemicals Europe GmbH), DNeasy Blood and Tissue (Qiagen®) or the QIAmp DNA Investigator Kit (Qiagen®).

#### 2.2.2 Mitochondrial DNA sequencing

We amplified 473 base pairs of mitochondrial control region (d‐loop) for each individual following the protocol from (Durka et al., 2005). Negative controls were included throughout and analysed in parallel with the samples. We used Geneious Prime 2019 (https://www.geneious.com) to trim and quality check the resulting sequences.

#### 2.2.3 Reduced representation sequencing

We produced a ddRAD library using a modified version of (Peterson et al., 2012) and the restriction enzymes *Sph*I and *Sbf*I (see Brown et al., 2016; Manousaki et al., 2015). We barcoded each sample using a 5 or 7 bp barcode on each end of the fragment, and then pooled and size selected the fragments (400–700 bp). We sequenced samples across two libraries and included negative and positive controls to enable quality control of the experimental process (plate 1: one within‐plate positive control and one negative control; plate 2: two within‐plate positive controls and one negative control; across plates 1 and 2: one between‐plate control). We quantified the resulting libraries using the Qubit dsDNA BR Assay (ThermoFISher Scientific) and sequenced each library on a single lane using 150 bp paired‐end sequencing on a single lane on a HiSeq 4000 System (Illumina, Inc).

#### 2.2.4 SNP calling and filtering

We demultiplexed reads using the *process_radtags* module of the Stacks 2.52 bioinformatics package (Catchen et al., 2013; Rochette et al., 2019). We used default parameters for removing reads with uncalled bases, discarding reads with low quality scores, and trimmed reads to 135 bp following an assessment of read quality using FastQC (Andrews, 2010).

There is currently no publicly available genome for the Eurasian beaver. Instead, we performed reference‐guided SNP identification using the genome of the closely related American beaver (*C. canadensis*) C.can_genome_v1.0_HiC (https://www.dnazoo.org/assemblies/Castor_canadensis). This genome assembly combines the draft assembly C.can_genome_v1.0 (GCF_001984765.1) (Lok et al., 2017) with Hi‐C assembly (Dudchenko et al., 2017, 2018). We used a custom Snakemake (Köster & Rahmann, 2012) pipeline developed by Dicks et al. (2023) for both mapping and SNP calling using the following tools. We mapped our demultiplexed reads against this reference genome using BWA v0.7.17 (Li & Durbin, 2009), and removed unmapped reads using Samtools v1.10 (Li et al., 2009). We excluded samples with fewer than 150,000 mapped reads from downstream analysis. Following mapping, we called SNPs in STACKS v2.52 (Rochette et al., 2019) using the marukilow model with default parameters.

Once SNPs were called, we filtered low‐quality loci and individuals using VCFtools (Danecek et al., 2011). Initially, we excluded genotypes if the minimum read depth was less than 5, and we excluded SNPs if the minimum mean read depth was less than 15X. We also excluded SNPs if the minor allele count was less than 3 to minimise PCR errors by ensuring all alleles were detected in a minimum of two samples. Subsequently, we filtered SNPs and individuals in a stepwise manner as recommended by O'Leary et al. (2018), whereby we excluded SNPs with >70% missing data, then individuals with >75% missing genotypes, and finally filtered both SNPs and individuals to retain only those with at least 80% genotyping rate.

After filtering, we assessed deviation from Hardy Weinberg equilibrium as either heterozygote excess or deficit within each population using an Exact Test (Wigginton et al., 2005) implemented within PLINK 1.9 (Chang et al., 2015). The Hardy Weinberg equilibrium exact test *p*‐values were adjusted for multiple testing using the Benjamini and Yekutieli (2001) method. We filtered out SNPs that were significantly out of Hardy Weinberg equilibrium in more than two populations to identify SNPs that were more likely to suffer from technical artefacts rather than SNPs demonstrating biologically meaningful signatures. We also calculated linkage disequilibrium (*r* ^2^) using PLINK 1.9 and considered two SNPs to be in linkage disequilibrium if they had a *r* ^2^ above 0.8 in more than two populations. We excluded the SNP with the lowest genotyping rate in each pair.

We assessed relatedness among the samples using the KING‐robust estimator (Manichaikul et al., 2010) implemented with NGSRelate v2 (Hanghøj et al., 2019) to confirm repeatability of positive controls and detect sample mix‐ups (following Duntsch et al., 2022). All within‐ and between‐plate positive controls revealed high relatedness (KING >0.48, where KING kinship coefficients of 0.354–0.5 correspond to duplicates; Manichaikul et al., 2010). Additionally, we identified one set of three samples and two sets of two samples with high similarity (KING > 0.49), which were subsequently each determined to represent the same individual, corroborated by field data and mitochondrial haplotypes. In all three cases, only the sample with the highest genotyping rate was retained. For positive controls, we retained only the sequencing repeat with the highest genotyping rate. The final set of SNPs and samples were quality assessed using VCFtools (Danecek et al., 2011), and results are shown in File S2.

Choice of SNP calling methodology is known to affect downstream population genetics analyses (Shafer et al., 2016). While Eurasian beavers and North American beavers are sister species, they have different numbers of chromosomes (Lavrov, 1973). To assess whether using the genome of North American beaver as a reference for SNP calling had created any bias, we repeated all our analyses via de novo SNP calling within STACKS v2.52 using the *denovo_map.pl* pipeline, selecting m = 5 M = 2 n = 2 following parameterisation (see Supplementary methods, Table S2, Figures S1, S2).

### 2.3 Population genetic analyses

For all population genetic analyses, we divided our data into six “populations”: Norwegian reference population, Bavarian reference population, Knapdale trial (pre‐reinforcement), Tayside reference population, beavers translocated to Knapdale during reinforcement (referred to collectively as “reinforcement” group), and Knapdale current (post‐reinforcement). Note, the “Knapdale current” grouping contains some individuals that are also present in the “Knapdale trial” and “reinforcement” groups, but excludes individuals from those groups that are known to have died before the 2019 sampling effort. Individuals that were born in Knapdale only appear in the “Knapdale current” group. These groups were designed to assess changes driven by the various translocations that have taken place for the overall Scottish beaver population.

#### 2.3.1 Mitochondrial DNA

We aligned our d‐loop haplotype sequences using the Geneious Alignment function in Geneious Prime 2019. We combined our haplotypes with those described in Senn et al. (2014) (also see Table S3) and produced a haplotype network using Popart 1.7 (Leigh & Bryant, 2015) using the median joining network methods with epsilon set at zero.

#### 2.3.2 Nuclear DNA

We used HIERfstat v0.5‐7 in R (Goudet, 2005) to calculate observed heterozygosity (*H*_o_), gene diversity (*H*_s_), allelic richness standardised to 11 samples (Ar), observed fixation index (*F*_IS_), and the number of fixed loci per population. We conducted AMOVAs and obtained pairwise *F*_ST_ values and significance probabilities in Arlequin v3.5 (Excoffier & Lischer, 2010).

To examine population structure, we conducted a principal component analysis (PCA) in adegenet v2.1.2 (Jombart, 2008; Jombart & Ahmed, 2011) using Radiator (Gosselin et al., 2020) for file conversion. As population structure is best examined using several lines of evidence, we also ran a Bayesian estimation of population structure in STRUCTURE 2.3.4 (Pritchard et al., 2000), which we parallelised using ParallelStructure v1.0 (Besnier & Glover, 2013). We used the admixture model, with the correlated allele frequencies model, lambda set to 1, and a burn in of 500,000, followed by 1,000,000 MCMC iterations. We performed five replicate runs for each value of the number of clusters (*K*) from one to six. We used the CLUMPAK web interface to summarise replicates and visualise the results (Kopelman et al., 2015). We examined the most likely number of clusters (*K*) using the delta *K* (Δ*K*) method described in (Evanno et al., 2005), implemented in STRUCTURE HARVESTER (Earl & vonHoldt, 2012) and via visual assessment of the STRUCTURE‐generated bar plots. We note that STRUCTURE and similar programmes are not designed to determine numbers of populations per se, but as an exploration of genetic structuring (Pritchard et al., 2007), and thus results from the Evanno method should be used with caution.

Finally, we estimated genetic clustering in ADMIXTURE version 1.3.0 (Alexander et al., 2009), with 10,000 iterations for each value of *K* (1–6). We visualised our results from ADMIXTURE using CLUMPAK web interface (Kopelman et al., 2015).

We estimated relatedness within populations rather than inbreeding due to the limitations of the tools currently available for robust estimations of inbreeding from reduced representation data. Estimating identity by descent from genetic markers usually relies on either the assumption that individuals belong to a homogenous population with random mating (as in PLINK; Purcell et al., 2007) or the existence of a reference dataset to estimate admixture proportions for each individual sampled (as in REAP; Thornton et al., 2012). Similarly, the majority of marker‐based relatedness estimator algorithms assume a homogenous population, and population structure will tend to bias results, inflating relatedness values for individuals from the same population (Manichaikul et al., 2010). To examine relatedness within the populations in this study, we produced pairwise relatedness estimates using the KING‐robust allele‐frequency free estimator of relatedness (Manichaikul et al., 2010), calculated in NGSRelate v2 (Hanghøj et al., 2019). The KING‐robust estimator does not rely on accurate estimations of allele‐frequencies and is therefore robust to small sample sizes and populations that violate the assumptions listed above (Manichaikul et al., 2010). KING‐robust does, however, produce downwardly biased estimates of relatedness when pairs of individuals have different population ancestry (Conomos et al., 2016; Thornton et al., 2012) and this should be taken into account when interpreting results. We assessed familial relationships between individuals using the method described by Waples et al. (2019) whereby KING‐robust kinship and R0 are plotted against R1. R0, R1 and KING‐robust are all statistics summarising patterns of identity‐by‐state across genotypes between two individuals. Relatedness categories can be inferred through visualisation of the relationships between KING‐robust and R1, and between R0 and R1. We note, however, that separation in the expected ranges for familial relationships is not predicted to remain distinct when inbred individuals are included (Waples et al., 2019). To assess the reliability of the kinship estimates, we compared them to some known (from observational data) relationships among the Norwegian Knapdale beavers.

## 3 RESULTS

### 3.1 Sequencing results

Prior to filtering, our reference‐based SNP calling pipeline produced a dataset containing 128 samples and 15,563 SNPs. During filtering, seven samples were removed due to genotyping rates below the selected thresholds and nine due to being samples from populations not investigated here. After confirming high levels of reproducibility between positive controls, we excluded one replicate from each of the four sample pairs. Checking for duplicate individuals resulted in the detection of four individuals sequenced across two samples each, and one of each pair of samples being excluded. This resulted in a dataset of 104 unique individuals genotyped at 2183 SNPs. Following filtering for HWE and LD, the number of SNPs was reduced to 2031.

### 3.2 Genetic diversity

**FIGURE 2:**
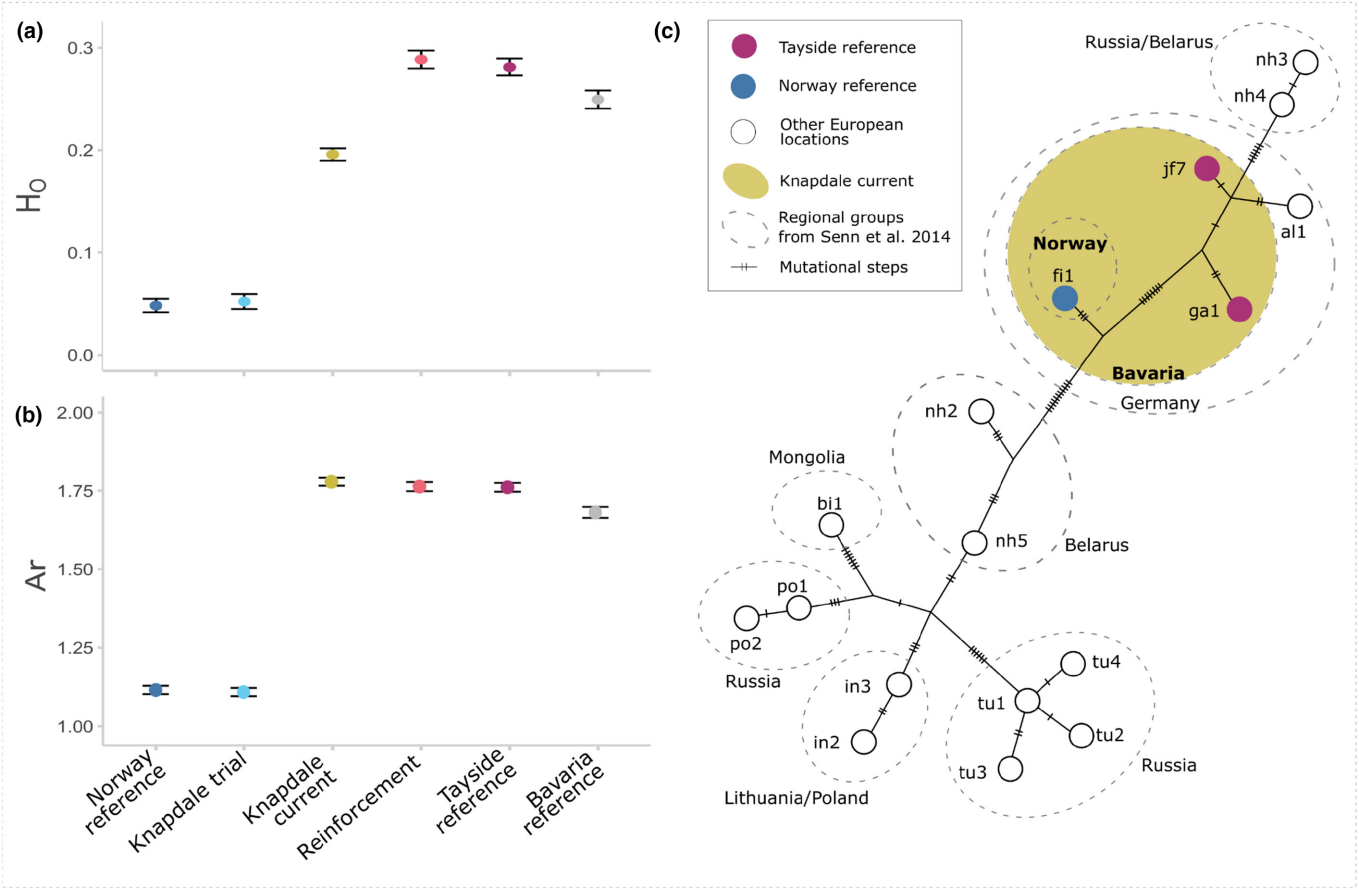
Genetic diversity at nuclear and mitochondrial markers for beavers in Scotland. (a) Observed heterozygosity for each of the beaver “populations” in our sample set estimated using 104 individuals genotyped at 2031 SNPs. (b) Allelic richness for each of the beaver “populations” in our sample set estimated using 104 individuals genotyped at 2031 SNPs. In both (a) and (b), error bars represent 95% confidence intervals. (c) Mitochondrial haplotypes based on 473 base pairs of the d‐loop in the mitochondrial control region. The haplotypes seen in the current Knapdale population (Norway reference (blue) and Tayside reference (magenta)) are put into the context of the wider contemporary Eurasian beaver population (modified from data in Senn et al., 2014).

Both our mitochondrial and nuclear DNA data illustrate higher genetic diversity in the Bavarian reference than the Norwegian reference beaver populations (Table 1, Figure 2). Bavarian reference beavers exhibited three mtDNA d‐loop haplotypes, two of which (ga1 and JF7) are distinct from the single haplotype seen in the Norwegian reference population and shared with Bavarian reference animals (fi1). Bavarian reference beavers also exhibited significantly higher heterozygosity and allelic richness, and substantially fewer fixed nuclear loci than their Norwegian counterparts. Both reference populations were in Hardy–Weinberg equilibrium.

**TABLE 1:** Genetic diversity summary for Scotland's beavers and their source populations based on 104 individuals genotyped at 2031 SNPs.

	*N*	*H*_o_	*H*_s_	Ar (*n* = 11)	*F*_IS_	Fixed loci (prop.)	KING‐robust
Norway reference	29	0.048	0.047	1.115	−0.009	1755 (0.86)	0.035
(95% CI)		(0.042–0.055)	(0.041–0.052)	(1.101–1.128)	(−0.051 to 0.032)		(0.024 to 0.046)
Knapdale trial	11	0.052	0.045	1.108	−0.132	1793 (0.88)	0.159
(95% CI)		(0.045–0.059)	(0.039–0.051)	(1.095–1.121)	(−0.177 to −0.087)		(0.134 to 0.184)
Knapdale current	29^a^	0.196	0.298	1.778	0.248	96 (0.047)	0.028
(95% CI)		(0.190–0.202)	(0.291–0.305)	(1.766–1.791)	(0.233 to 0.263)		(0.009 to 0.048)
Reinforcement	19	0.289	0.298	1.763	0.034	224 (0.11)	−0.025
(95% CI)		(0.280–0.297)	(0.290–0.306)	(1.749–1.777)	(0.022 to 0.048)		(−0.046 to −0.004)
Tayside reference	22	0.281	0.295	1.761	0.047	177 (0.09)	−0.032
(95% CI)		(0.273–0.290)	(0.287–0.302)	(1.747–1.775)	(0.036 to 0.059)		(−0.045 to −0.018)
Bavaria reference	17	0.250	0.273	1.680	0.075	426 (0.21)	−0.079
(95% CI)		(0.241–0.258)	(0.264–0.281)	(1.663–1.698)	(0.061 to 0.088)		(−0.094 to −0.064)

Abbreviations: Ar, allelic richness; CI, confidence intervals; *F*_IS_, fixation index; *H*_o_, observed heterozygosity; *H*_s_, gene diversity.

^a^ Note that this category includes 6 individuals born in Knapdale and 23 individuals that are also included in other categories (7 in Knapdale trial, 16 in Reinforcement).

The Knapdale trial beavers showed no discernible difference in genetic diversity compared to their source population in Norway (based on 95% CIs, Table 1, Figure 2). The Tayside reference beavers actually showed slightly, but significantly higher heterozygosity and allelic richness compared to their source population in Bavaria. Correspondingly, genetic diversity in the Tayside reference population was higher than in the Knapdale trial (pre‐reinforcement) population. No changes to mtDNA haplotype presence in beavers in Britain were detected, with the Knapdale trial beavers possessing the same single d‐loop haplotype as the Norwegian reference population (fi1), and the Tayside reference beavers showing the two distinct haplotypes from the Bavarian reference population (ga1 and JF7), but not the haplotype that is also seen in the Norwegian reference population (fi1). The Knapdale trial population exhibited a significantly negative *F*_IS_ value, demonstrating an excess of heterozygotes compared to that expected under Hardy–Weinberg equilibrium, while the Tayside reference beavers showed a small, marginally significant, positive *F*_IS_ value, demonstrating a slight deficit of heterozygotes.

The beavers used for the reinforcement translocation into Knapdale showed no difference in genetic diversity compared to the Tayside reference population at either mitochondrial or nuclear DNA (based on 95% CIs and haplotypes present, Table 1, Figure 2, Table S1). Introducing these individuals into Knapdale has caused significant changes in genetic diversity in the overall Knapdale population, increasing the number of mtDNA haplotypes present from one to three, significantly increasing heterozygosity and allelic richness, and substantially reducing the number of fixed loci present in the post‐reinforcement Knapdale population (Table 1, Figure 2). Heterozygosity and allelic richness in the post‐reinforcement Knapdale population remained lower than in the Tayside or the Bavarian reference populations, but mtDNA d‐loop haplotype diversity was greater in Knapdale than in the Norway reference population and equal to that in the Bavarian reference, and the number of fixed loci in Knapdale was lower than in either reference population. The current Knapdale population showed a significant deficit of heterozygotes, likely due to a Wahlund effect (see Section 4).

### 3.3 Genetic differentiation

**FIGURE 3:**
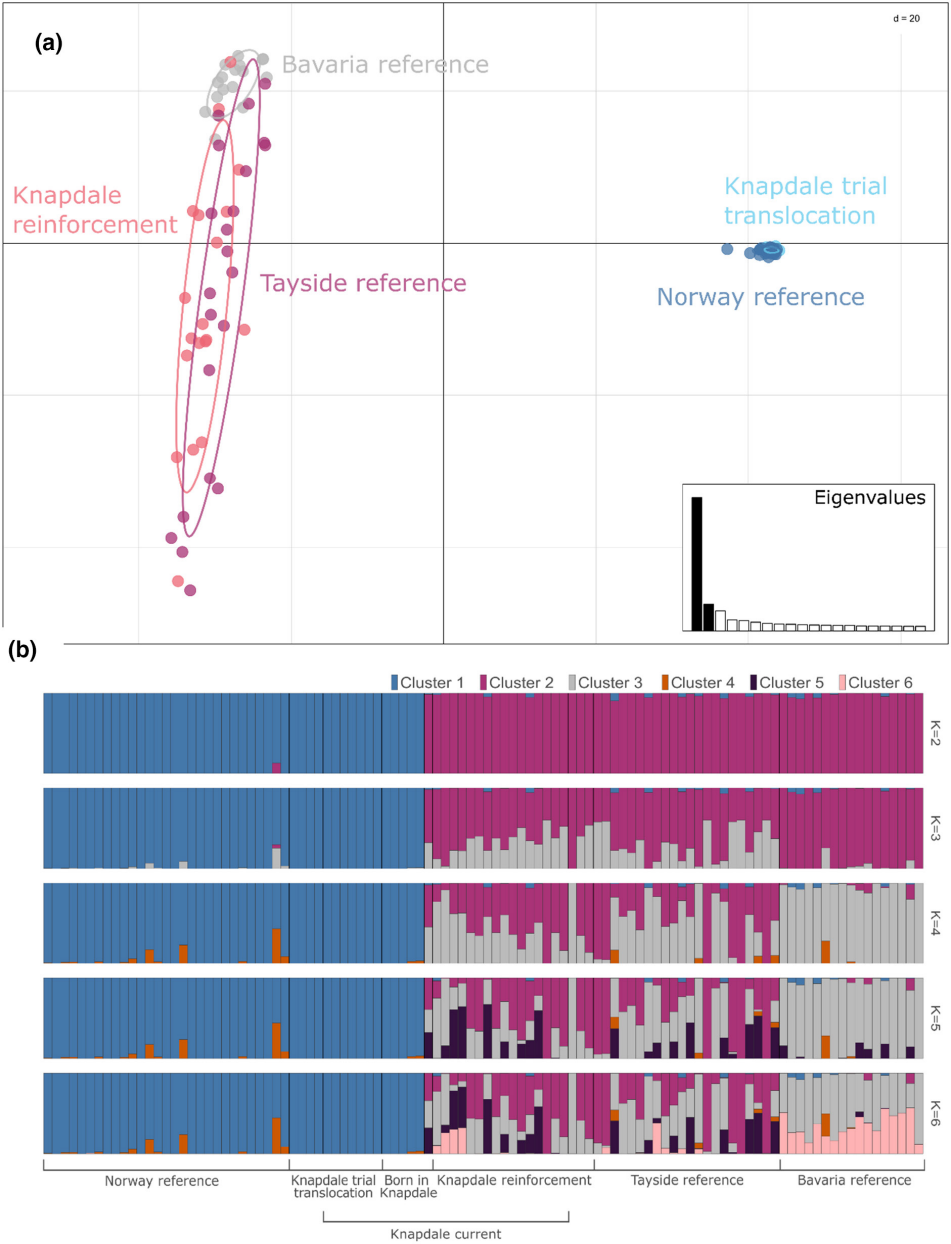
Population structure in Scotland's beavers and alignment to respective source populations based on 104 individuals genotyped at 2031 SNPs. (a) PCA analysis. (b) STRUCTURE analysis results showing outputs for *K* = 2–*K* = 6.

At the time of sampling there was no evidence of any admixture between trial (Norwegian stock) and reinforcement (Bavarian stock) animals in Knapdale (Figure 3). The Norwegian reference population and the Knapdale trial group were more genetically similar to one another than either was to any of the Bavarian groups, and vice versa. This pattern is demonstrated in the *F*_ST_ values (Table 2), PCA (Figure 3a), and Bayesian analyses (Figure 3b).

**TABLE 2:** *F*_ST_ values between sample sets of beavers in this study.

	Norway reference	Knapdale trial	Reinforcement	Knapdale current	Tayside reference	Bavaria reference
Norway reference	0					
Knapdale trial	0.0271*	0				
Reinforcement	0.5968*	0.5089*	0			
Knapdale current	0.2940*	0.2235*	0.0977*	0		
Tayside reference	0.5755*	0.4915*	0.0034	0.1818*	0	
Bavaria reference	0.6283*	0.5403*	0.0603*	0.1413*	0.0559*	0

* Statistically significant values (*α* = 0.05).

In the PCA, PC1 clearly separated the beaver samples into Norwegian and Bavarian origin animals, with PC2 highlighting the small amount of additional genetic diversity seen in Tayside versus the Bavarian reference sample. The results from STRUCTURE and ADMIXTURE were in strong agreement (Figure 3, Figure S4) and support the PCA, showing a clear division between Norwegian and Bavarian animals, with an additional cluster being introduced at *K* = 3 that was only seen in Tayside animals, and not in the Bavarian reference population.

### 3.4 Inbreeding and kinship

**FIGURE 4:**
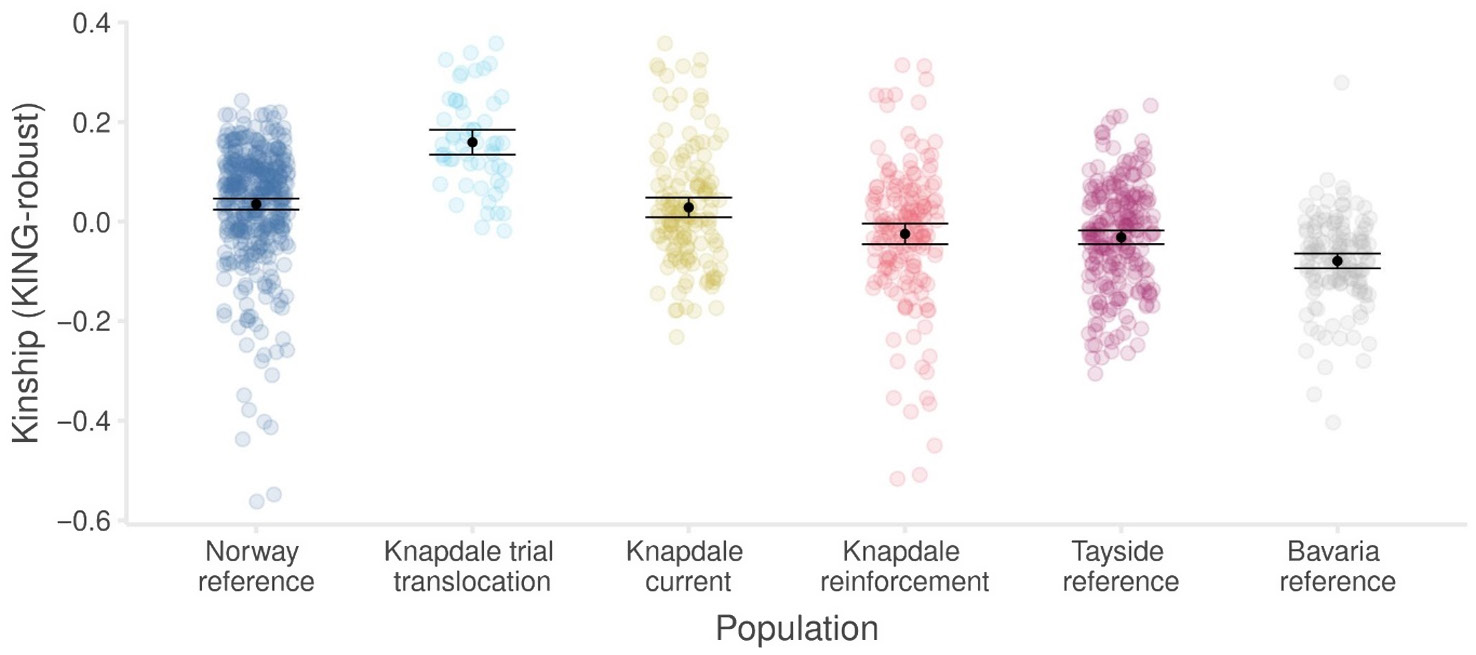
Relatedness in Scotland's beavers and their respective source populations based on 104 individuals genotyped at 2031 SNPs. Pairwise KING‐robust kinship estimates are shown as coloured points, mean population estimates as black points, and black error bars are 95% confidence intervals.

The kinship analyses suggested that average relatedness in the Norwegian reference population was significantly greater than that in the Bavarian reference population and that relatedness in the Knapdale trial population was significantly higher again (Figure 4). The reinforcement translocation into Knapdale had reduced the average relatedness in the population significantly, rendering it similar to the Norwegian reference population (Figure 4). The magnitude of this reduction should, however, be treated with caution due to the tendency of the KING‐robust estimator to downwardly bias relatedness estimates in dyads involving individuals with differing population ancestry (see Section 4). In our comparison of known parent‐offspring, full sibling, and second‐degree relative dyads, the majority of these relationships fell as expected in relation to one another. Further, the Waples et al. (2019) method highlighted a potential mismatch in the observational data in the relationship of two of the beavers in the particularly inbred Knapdale family group where a suspected grandmother and grandson may actually have been mother and son, and the son's suspected mother was actually his full sibling (Figure S5).

## 4 DISCUSSION

The population genetics of Eurasian beavers in mainland Europe has been profoundly affected by a history of hunting‐induced population bottlenecks followed by a mix of natural and human‐assisted re‐colonisation (Halley et al., 2021; Horn et al., 2014; Nolet & Rosell, 1998; Senn et al., 2014). Genetic diversity in European beavers remains low compared to historic levels, despite recent population expansions (Horn et al., 2014). Here, we demonstrate that this history has consequences for the reintroduced population of beavers in Scotland, which has been founded with individuals from populations with two very different histories – Norway and Bavaria. These contrasting histories have created opportunities to reinforce the Knapdale beaver population following its establishment with a handful of individuals from the relatively genetically impoverished Norwegian population. What remains to be seen is whether this genetic foundation is enough to create a sustainable long‐term future for beavers in Scotland as a whole and, indeed, the rest of Britain or whether further genetic rescue attempts will be required.

### 4.1 What (if any) genetic diversity was lost when beavers were translocated from Norway to Knapdale, and Bavaria to Tayside?

The translocations of beavers from Norway to Knapdale and Bavaria to Tayside seemingly captured all the genetic diversity detected here using reduced representation sequencing from each of their respective source populations. This is relatively intuitive as, the lower the genetic variation in the source population, the fewer individuals are required to capture that diversity. Norwegian beavers exhibit some of the lowest genetic diversity of any Eurasian beaver population yet sampled, a relic of the population being reduced to around 60–120 individuals in 1880 (Collett, 1897). That diversity was seemingly captured in the 16 beavers translocated to Knapdale in 2009/10, but the low genetic diversity seen in Norway meant that Knapdale was founded with correspondingly low diversity. With just nine individuals remaining by the end of the Scottish Beaver Trial, the population was also at risk of inbreeding (mating between relatives) due to small population size and the population features at least one father‐daughter pairing. Rodents are often cited as being immune to the harmful effects of inbreeding, but both laboratory and empirical studies suggest otherwise (e.g., Jimenez et al., 1994; Lacy et al., 1996; Lacy & Horner, 1997; Olson et al., 2012; Ross‐Gillespie et al., 2007). The current agreed thinking is that beaver populations are best established using admixed individuals (Senn et al., 2014), with the risk of inbreeding outweighing that of outbreeding (Frankham et al., 2011) and evidence of hybrid vigour in admixed beaver populations (Halley, 2011).

Bavarian beavers exhibit more genetic diversity than their Norwegian counterparts and are known to be genetically admixed following reintroductions into the area from France, Belarus/Lithuania/Poland, Russia, and Norway (Nolet & Rosell, 1998; Senn et al., 2014). Only a small amount of Norwegian genetic material has been detected in reference samples from Bavaria, suggesting that Norwegian animals may not have contributed greatly to this population and making the Bavarian and Norwegian populations genetically distinct (Senn et al., 2014). The exact number of founders moved from Bavaria (and potentially elsewhere) to Tayside is uncertain due to the unauthorised nature of this release, but genetic data based on a relatively small sample of 43 individuals suggest a modest release of around three family groups (Campbell‐Palmer et al., 2020; McEwing et al., 2015). Regardless of how many founders were used, they were sufficient to capture the known mitochondrial and the majority of nuclear diversity present in the Bavarian population, with the exception of any Norwegian genetic material (which was not detected in the Tayside sample). In fact, Tayside beavers exhibit very slightly higher nuclear genetic diversity than their Bavarian counterparts. This is due to the presence of an additional nuclear genetic signature that is not present in the original Bavarian reference sample, which could be due to lack of representation in the reference sample itself (*n* = 48) or could raise further questions about the origins of the illegal Tayside release.

A recent study using a different genetic data set to the original assessment of the Tayside population (Campbell‐Palmer et al., 2020 vs. McEwing et al., 2015) corroborated the most likely origins of the individuals in Tayside as Bavarian. However, that study also identified the presence of a single individual with origins more closely correspondent with reference samples from river systems that span Lithuania, Poland, Belarus, and Russia (Campbell‐Palmer et al., 2020). Whether this is due to the translocation of an individual brought in separately from one of those river systems, an unadmixed individual remaining within Bavaria since the reintroductions between 1966 and 1989 (Halley & Rosell, 2003; Nolet & Rosell, 1998), or a secondary illegal release using beavers from another origin is currently unknown. Regardless, the increased sample set and extensive genetic data used in our study reveals a pervasive genetic signature within Tayside that is not observed in our Bavarian reference samples, and that has now also been translocated to Knapdale.

### 4.2 What (if any) genetic diversity has been gained by translocating Bavarian‐stock beavers into Knapdale?

Due to the discrete genetic origins of the beavers used in the original Knapdale trial (Norwegian) and in the reinforcement (majority Bavarian), the reinforcement translocation has significantly increased the genetic diversity present in the overall Knapdale population. The reinforcement group of 21 beavers translocated to Knapdale captured the known genetic diversity from Bavaria and additional diversity from Tayside, giving the best possible chance for improvements to genetic diversity in Knapdale. The overall Knapdale population is now significantly more heterozygous than it was before (although remains significantly less heterozygous than the Tayside population) and exhibits more mitochondrial and allelic diversity than either the pre‐reinforcement Knapdale Norwegian or the Tayside population in isolation.

Perhaps unsurprisingly, translocating genetically distinct beavers into Knapdale has also reduced the overall kinship coefficient of the Knapdale beaver population. However, the significant decrease in average relatedness seen following the reinforcement translocation into Knapdale should be treated with caution as the KING estimator tends to downwardly bias estimates of relatedness between pairs of individuals from different ancestral populations (Conomos et al., 2016; Thornton et al., 2012) and there are many of these dyads in the reinforced Knapdale population. Meaningful reductions in relatedness will hopefully result from admixture between the beavers of Norwegian and Bavarian ancestry (see below). However, the degree to which this admixture reduces the average kinship in Knapdale will depend on the persistence of inbreeding in the current main Knapdale Norwegian stock family, which comprises offspring from an original translocated pair, and then, following the death of the female in that pair, the offspring of the original male and his daughter (Figure S5). What we now believe (based on data presented in this study) to be the son of the original pair and a daughter of the father‐daughter pairing are thought to have formed a pair and produced offspring, creating a highly inbred family matrix that could cause average relatedness in the Knapdale population to remain high on average (Figure S5).

Taken together, these data paint a positive genetic outlook for the Knapdale beaver population, which should now have a greater ability to adapt and survive through changing circumstances (Allendorf, 1986; James, 1970; Olazcuaga et al., 2023; Robertson & Waddington, 1997), and a chance to avoid the potentially negative consequences of inbreeding. Whether or not this potential is realised is contingent on beavers of Norwegian origin and Bavarian origin cross‐breeding in Knapdale and whether the Knapdale beaver population ever becomes connected with the wider Scottish population (see below).

### 4.3 Is there any evidence of admixture between Norwegian and Bavarian‐stock beavers within the Knapdale population at this point?

The genetic data presented here, combined with field observations (Dowse et al., 2020) suggest that, at the end of the Scottish Beavers reinforcement project, there had been no admixture between Norwegian and Bavarian beavers in Knapdale. There are no individuals in our sample that exhibit any kind of admixed genetic profile in either Structure or PCA analyses. The Knapdale population is also significantly out of Hardy–Weinberg equilibrium due to an excess of homozygotes, suggesting a Wahlund effect, which occurs when genetically distinct populations are grouped together in the same sample (Wahlund, 1928). Field data from the Knapdale site collected throughout the reinforcement and in the year post these 2 years of translocations showed Norwegian and Bavarian beavers breeding successfully, but pairing up within their own genetic lineages (Dowse et al., 2020).

The reinforcement translocations were always unlikely to produce admixture within the 3‐year duration of the reinforcement project. Before the reinforcement, Knapdale was home to at least two established pairs/families of Norwegian origin beavers from the original translocations in 2009/10. As mentioned above, one of these families is highly inbred. The other is a pair that is not known to have any surviving offspring. The majority of beavers translocated into Knapdale were moved as known/suspected family groups or released together into locations in the hope they would be less likely to immediately disperse from a site (Campbell‐Palmer & Jones, 2014). Eurasian beavers are relatively monogamous and tend to form pairs that last many generations, if not their entire reproductive lives (Syrůčková et al., 2015; Wilsson, 1971). The most likely scenario for admixture will arise from the offspring of Norwegian and Bavarian single‐origin pairs dispersing and pairing up. Beavers take at least 2 years to reach sexual maturity and tend to remain within their family groups for an average of 3.5 years (although this can be extended up to 7 years at high population densities) (Hartman, 1997; Mayer et al., 2017), so it may be several years before genetic signatures of admixture are detectable in the Knapdale population. Such challenges are not uncommon when attempting to encourage admixture via translocation in species with complex social biology and behaviour (Miller et al., 2009; Robledo‐Ruiz et al., 2022; Thavornkanlapachai et al., 2019). Follow up surveys in 2024 and 2029 (5 and 10 years after the end of the reinforcement project) would be useful for long‐term genetic monitoring of this population. Whether such admixture would lead to genetic rescue (population growth following the introduction of new genetic material; Tallmon et al., 2004) remains to be seen.

### 4.4 The potential importance of Knapdale's beavers

If admixture between Norwegian and Bavarian lines does occur in the Knapdale population, these animals will occupy an interesting position in the wider Eurasian beaver landscape. In the event of incomplete admixture, the Knapdale beaver population will have a different genetic profile to any Eurasian beaver population yet studied. Even with complete admixture, the Knapdale population would be an important reservoir of genetic material, and combinations of that material, currently not found anywhere else in Britain.

The Knapdale population is small and isolated by design. Intended as a trial for wider beaver reintroductions to Scotland, Knapdale was selected as a site that would be relatively impermeable to dispersal, allowing the trial to be contained and controlled in case of unintended consequences (Campbell‐Palmer & Jones, 2014). However, this now renders Knapdale an isolated population which, even with new genetic material, will be subject to genetic erosion over time (Falconer & Mackay, 1996, pp. 66; Wright, 1931) and a propensity towards inbreeding in the longer term. All previous genetic studies of other beavers in Britain suggest other populations are of Bavarian origin (with the exception of the potential small number of animals from the Polish/Lithuanian/Belarusian/Russian river systems mentioned above) and they greatly outnumber the Knapdale population (roughly 68:1; Campbell‐Palmer et al., 2021; Dowse et al., 2020).

The Tayside population is spreading west towards Knapdale and it is possible that these two populations might eventually meet (although this is unlikely to happen without assistance as mentioned above). The Scottish Government has now sanctioned additional translocations of beavers to form new populations in previously unoccupied areas of Scotland (subject to license approval) (IUCN/CPSG, 2022), and some of these new populations could also expand towards Knapdale and/or more beavers could be translocated from Tayside into Knapdale. This would remove the issue of Knapdale being isolated, but it could also lead to Tayside genetic material dominating the Knapdale population, risking the loss of Norwegian and potential admixed genetic diversity that is currently not found elsewhere in Britain. This diversity may, at some point, become important for the long‐term persistence of the British beaver population and so should be preserved.

With the exception of Knapdale and the small number of potential Polish/Lithuanian/Belarussian/Russian beavers discussed above, the other sampled British beaver populations (Beauly and the River Otter in Devon) are descended from Bavarian beavers (Campbell‐Palmer et al., 2020). While Tayside beavers form the majority of the Scottish population, they have also been used as a source of translocations to fenced populations in England as part of a gradual move towards reintroducing beavers there (Dowse et al., 2020). The unauthorised release of beavers that led to the River Otter population in Devon, England, also used Bavarian beavers (Campbell‐Palmer et al., 2020). As Britain is, itself, an island, there are no natural routes for geneflow between beavers in Britain and mainland Europe. There are, currently, no plans for additional translocations of beavers from Europe into Great Britain, but new genetic assessments of the English beaver population are assessing the potential of such translocations (Ritchie‐Parker et al, in preparation).

Without additional translocations of beavers from mainland Europe into Great Britain, the British population of beavers will be subject to genetic erosion and, depending on how rapidly the population expands and how connected it is, could also be subject to inbreeding. It is also worth bearing in mind that beavers in mainland Europe have already suffered significant genetic erosion due to population bottlenecks caused by hunting for the fur trade (Senn et al., 2014) and that this low genetic diversity persists despite rapid population expansions (Horn et al., 2014); beavers in Britain represent a subset of these previously bottlenecked populations. This situation is sub‐optimal for long‐term population persistence of beavers in Britain. If Knapdale becomes a source of unique genetic material in Great Britain, it could help to ameliorate wider genetic issues for British beavers, both via reinforcement translocations and as a source for establishing new local populations. Maintaining this potentially important genetic mix in Knapdale will require careful management and monitoring, plans for which are incorporated in the recently published national strategy for beavers in Scotland (IUCN/CPSG, 2022).

### 4.5 Genetic monitoring to fully assess the success of translocations

This study illustrates the usefulness of genetic data collected over time in assessing the successes and shortcomings of a translocation process. It also presents an example of a system where, as is so often the case, genetic analyses timelines did not marry with the timelines of conservation action on the ground. This is perhaps not surprising given that genetic data are often missing from conservation management planning and policy in general (Cook & Sgrò, 2017; Pierson et al., 2016), but the work here sets the stage for continued inclusion of genetic data in the planning of further beaver translocations in Britain, and conservation translocations in general. The reinforcement actions undertaken for beavers in Knapdale following a proper genetic assessment have the potential to lead to genetic rescue and seem to have improved this population's chances of long‐term persistence, but it would undoubtedly have been better to have founded the population with that genetic knowledge in hand in the first place.

Numerous studies have used genetic tools to uncover the effects of a translocation after the fact and, in the process, demonstrate the importance of integrating long‐term genetic monitoring into reintroductions from their initiation (e.g., Dicks et al., 2023; Flesch et al., 2020; Ogden et al., 2020; Ramstad et al., 2013; Shapcott et al., 2009; Taylor, Colbourne, et al., 2017; White, Ottewell, et al., 2020). Increasingly, studies that review and highlight the genetic issues with previous translocation programs make recommendations for future efforts (e.g., White, Thomson, et al., 2020), or use these data to create predictive models that can be integrated into the adaptive management of translocated populations (e.g., Pacioni et al., 2018, 2020) or a spatial framework for translocation decision‐making (e.g., Norman & Christidis, 2021). In other programs, genetic data are now fully integrated into conservation translocation planning and species conservation management in general; the Tasmanian Devil Tools and Tech programme and the Saving Wildcats breeding for release programme are good examples of this (Hogg et al., 2017; Senn et al., 2019), but this is still the exception rather than the rule.

Previously, translocations occurred in the absence of genetic data because conservation genetics was not yet an established field, and the importance of genetic factors to population persistence was still being debated (Caughley, 1994; Craig, 1991; Jamieson et al., 2006). Recent discussions in the scientific literature continue to muddy the waters (Teixeira & Huber, 2021), despite being comprehensively rebuffed (Kardos et al., 2021; Willi et al., 2022). At this point, the evidence for genetic factors playing a role in long‐term species' persistence is clear (Kardos et al., 2021); the importance of genetics in species conservation is being advocated on an international policy level (Hoban et al., 2020, 2021), resulting in genetic diversity being integrated into the latest iteration of the Global Biodiversity Framework following COP15 (The Conference of the Parties to the Convention on Biological Diversity, 2022), genetic guidance is available for those conducting conservation translocations (Frankham et al., 2019; Ralls et al., 2018), and consideration of genetic factors is recommended in the IUCN translocation guidelines (IUCN Species Survival Commission, 2013). There is a clear need for a paradigm shift where, if possible, genetic data (or at the very least modelling) are considered as part of any species translocation plan, far in advance of a translocation taking place.

Often data on the source population (and alternative source populations) are simply not available at the time the translocation is being planned (as was the case for beavers in Scotland). For Scottish beavers, the genetic tools available at the time of the initial translocation in 2009 were suboptimal, with low microsatellite polymorphism hampering statistically powerful genetic assessments for the species (H. Senn, personal communication). The ddRAD study conducted on European beavers (Senn et al., 2014) was one of the first to use reduced representation sequencing for conservation purposes and thus European beaver genetics have been assessed with the best tools available at the time.

When robust genetic data are not available, should translocations be halted to avoid the need for later genetic rescue? This hardly seems practical given the timelines often involved in generating genetic data and the factors discussed above, but it is a clear demonstration of why building standard measures of genetic diversity into threat listing and conservation management planning is crucial if we are to shift from assessing genetic legacies and having to facilitate genetic rescue attempts for translocations to planning translocations more effectively. Conservation genetics is by no means a silver bullet to assure a species' persistence, and some translocations may always necessitate follow‐up genetic rescue attempts due to difficulties in sourcing founders for threatened species. However, it is acknowledged that effective conservation requires holistic solutions (Liu et al., 2015) and genetics is an inherently important part of managing the kinds of small populations that frequently result from translocation efforts (Ralls et al., 2018). The genetic tools to make translocations more effective exist and are becoming ever more precise and informative; we must use them proactively wherever possible if we are to manage species reintroductions in a holistic and effective manner. Genetic rescue can be a useful action to facilitate long‐term success of conservation translocations, but it does not have to be an inevitable necessity if genetic data are properly integrated into translocation planning from the start.

## CONFLICT OF INTEREST STATEMENT

The authors declare no conflict of interest.

## Supporting information


File S1

File S2

## Data Availability

Raw, demultiplexed ddRAD data for this study have been deposited at the NCBI short read archive (https://www.ncbi.nlm.nih.gov/sra) under the project accession PRJNA860774 and biosample accessions SAMN29862098–SAMN29862201. The filtered ddRAD genotype data, mitochondrial haplotype alignment, and scripts used for analyses are available on GitHub (https://github.com/RZSS‐WildGenes/Genetic_rescue_Scot_beavers) and on Dryad (https://doi.org/10.5061/dryad.z08kprrm0).

## References

[eva13629-bib-0001] Alexander, D. H. , Novembre, J. , & Lange, K. (2009). Fast model‐based estimation of ancestry in unrelated individuals. Genome Research, 19(9), 1655–1664. 10.1101/gr.094052.109 19648217 PMC2752134

[eva13629-bib-0002] Allendorf, F. W. (1986). Genetic drift and the loss of alleles versus heterozygosity. Zoo Biology, 5(2), 181–190. 10.1002/zoo.1430050212

[eva13629-bib-0003] Andrews, S. (2010). FastQC: A quality control tool for high throughput sequence data . http://www.bioinformatics.babraham.ac.uk/projects/fastqc/

[eva13629-bib-0004] Bellis, J. , Bourke, D. , Williams, C. , & Dalrymple, S. (2019). Identifying factors associated with the success and failure of terrestrial insect translocations. Biological Conservation, 236, 29–36. 10.1016/j.biocon.2019.05.008

[eva13629-bib-0005] Benjamini, Y. , & Yekutieli, D. (2001). The control of the false discovery rate in multiple testing under dependency. The Annals of Statistics, 29(4), 1165–1188. 10.1214/aos/1013699998

[eva13629-bib-0006] Berger‐Tal, O. , Blumstein, D. T. , & Swaisgood, R. R. (2020). Conservation translocations: A review of common difficulties and promising directions. Animal Conservation, 23(2), 121–131. 10.1111/acv.12534

[eva13629-bib-0007] Besnier, F. , & Glover, K. A. (2013). ParallelStructure: A R package to distribute parallel runs of the population genetics program STRUCTURE on multi‐core computers. PLoS One, 8(7), 1–5. 10.1371/journal.pone.0070651 PMC3726640 23923012

[eva13629-bib-0008] Biebach, I. , & Keller, L. F. (2010). Inbreeding in reintroduced populations: The effects of early reintroduction history and contemporary processes. Conservation Genetics, 11(2), 527–538. 10.1007/s10592-009-0019-6

[eva13629-bib-0009] Brown, J. K. , Taggart, J. B. , Bekaert, M. , Wehner, S. , Palaiokostas, C. , Setiawan, A. N. , Symonds, J. E. , & Penman, D. J. (2016). Mapping the sex determination locus in the hāpuku (*Polyprion oxygeneios*) using ddRAD sequencing. BMC Genomics, 17(1), 448. 10.1186/s12864-016-2773-4 27286864 PMC4902995

[eva13629-bib-0010] Bubac, C. M. , Johnson, A. C. , Fox, J. A. , & Cullingham, C. I. (2019). Conservation translocations and post‐release monitoring: Identifying trends in failures, biases, and challenges from around the world. Biological Conservation, 238, 108239. 10.1016/j.biocon.2019.108239

[eva13629-bib-0011] Campbell‐Palmer, R. , & Jones, S. (2014). *The Scottish Beaver Trial: The story of Britain's first licensed release into the wild*. Final Report.

[eva13629-bib-0012] Campbell‐Palmer, R. , Puttock, A. , Needham, R. N. , Wilson, K. , Graham, H. , & Brazier, R. E. (2021). Survey of the Tayside area beaver population 2020‐2021 . NatureScot Research Report 1274.

[eva13629-bib-0013] Campbell‐Palmer, R. , Schwab, G. , Girling, S. J. , Lisle, S. , & Gow, D. (2015). Managing wild Eurasian beavers: A review of European management practices with consideration for Scottish application .

[eva13629-bib-0014] Campbell‐Palmer, R. , Senn, H. , Girling, S. , Pizzi, R. , Elliott, M. , Gaywood, M. , & Rosell, F. (2020). Beaver genetic surveillance in Britain. Global Ecology and Conservation, 24, e01275. 10.1016/j.gecco.2020.e01275

[eva13629-bib-0015] Catchen, J. , Hohenlohe, P. A. , Bassham, S. , Amores, A. , & Cresko, W. A. (2013). Stacks: An analysis tool set for population genomics. Molecular Ecology, 22(11), 3124–3140. 10.1111/mec.12354 23701397 PMC3936987

[eva13629-bib-0016] Caughley, G. (1994). Directions in conservation biology. Journal of Animal Ecology, 63(2), 215–244. 10.2307/5542

[eva13629-bib-0017] Chang, C. C. , Chow, C. C. , Tellier, L. C. A. M. , Vattikuti, S. , Purcell, S. M. , & Lee, J. J. (2015). Second‐generation PLINK: Rising to the challenge of larger and richer datasets. GigaScience, 4(1), 1–16. 10.1186/s13742-015-0047-8 25722852 PMC4342193

[eva13629-bib-0018] Collett, R. (1897). Bæveren i Norge, dens Utbredelsen og Levemaade. Bergens Museums Aarbog, 1, 1–139.

[eva13629-bib-0019] Conomos, M. P. , Reiner, A. P. , Weir, B. S. , & Thornton, T. A. (2016). Model‐free estimation of recent genetic relatedness. The American Journal of Human Genetics, 98(1), 127–148. 10.1016/j.ajhg.2015.11.022 26748516 PMC4716688

[eva13629-bib-0020] Cook, C. N. , & Sgrò, C. M. (2017). Aligning science and policy to achieve evolutionarily enlightened conservation. Conservation Biology, 31(3), 501–512. 10.1111/cobi.12863 27862324

[eva13629-bib-0021] Craig, J. L. (1991). Are small populations viable? Acta XX Congressus Internationalis Ornithologici, 4, 2546–2551.

[eva13629-bib-0022] Crowley, S. L. , Hinchliffe, S. , & McDonald, R. A. (2017). Nonhuman citizens on trial: The ecological politics of a beaver reintroduction. Environment and Planning A: Economy and Space, 49(8), 1846–1866. 10.1177/0308518X17705133

[eva13629-bib-0023] Danecek, P. , Auton, A. , Abecasis, G. , Albers, C. A. , Banks, E. , DePristo, M. A. , Handsaker, R. E. , Lunter, G. , Marth, G. T. , Sherry, S. T. , McVean, G. , & Durbin, R. (2011). The variant call format and VCFtools. Bioinformatics, 27(15), 2156–2158. 10.1093/bioinformatics/btr330 21653522 PMC3137218

[eva13629-bib-0024] Dicks, K. L. , Ball, A. D. , Banfield, L. , Barrios, V. , Boufaroua, M. , Chetoui, A. , Chuven, J. , Craig, M. , Faqeer, M. Y. A. , Garba, H. H. M. , Guedara, H. , Harouna, A. , Ivy, J. , Najjar, C. , Petretto, M. , Pusey, R. , Rabeil, T. , Riordan, P. , Senn, H. V. , … Gilbert, T. (2023). Genetic diversity in global populations of the critically endangered addax (*Addax nasomaculatus*) and its implications for conservation. Evolutionary Applications, 16(1), 111–125. 10.1111/eva.13515 36699120 PMC9850015

[eva13629-bib-0025] Dowse, G. , Taylor, H. R. , Girling, S. , Costanzi, J.‐M. , Robinson, S. , & Senn, H. (2020). Beavers in Knapdale: Final report from the Scottish Beavers Reinforcement Project . https://scottishwildlifetrust.org.uk/our‐work/our‐projects/scottish‐beavers/beavers‐in‐knapdale‐report/

[eva13629-bib-0026] Dudchenko, O. , Batra, S. S. , Omer, A. D. , Nyquist, S. K. , Hoeger, M. , Durand, N. C. , Shamim, M. S. , Machol, I. , Lander, E. S. , Aiden, A. P. , & Aiden, E. L. (2017). De novo assembly of the *Aedes aegypti* genome using Hi‐C yields chromosome‐length scaffolds. Science, 356(6333), 92–95. 10.1126/science.aal3327 28336562 PMC5635820

[eva13629-bib-0027] Dudchenko, O. , Shamim, M. S. , Batra, S. S. , Durand, N. C. , Musial, N. T. , Mostofa, R. , Pham, M. , St Hilaire, B. G. , Yao, W. , Stamenova, E. , Hoeger, M. , Nyquist, S. K. , Korchina, V. , Pletch, K. , Flanagan, J. P. , Tomaszewicz, A. , McAloose, D. , Pérez Estrada, C. , Novak, B. J. , … Aiden, E. L. (2018). The Juicebox Assembly Tools module facilitates de novo assembly of mammalian genomes with chromosome‐length scaffolds for under $1000. *BioRxiv* 10.1101/254797

[eva13629-bib-0028] Duntsch, L. , Brekke, P. , Ewen, J. G. , & Santure, A. W. (2022). Who are you? A framework to identify and report genetic sample mix‐ups. Molecular Ecology Resources, 22, 1855–1867. 10.1111/1755-0998.13575 34907643

[eva13629-bib-0029] Durka, W. , Babik, W. , Ducroz, J. F. , Heidecke, D. , Rosell, F. , Samjaa, R. , Saveljev, P. A. , Stubbe, A. , Ulevičius, A. , & Stubbe, M. (2005). Mitochondrial phylogeography of the Eurasian beaver *Castor fiber* L. Molecular Ecology, 14(12), 3843–3856. 10.1111/j.1365-294X.2005.02704.x 16202100

[eva13629-bib-0030] Earl, D. A. , & vonHoldt, B. M. (2012). STRUCTURE HARVESTER: A website and program for visualizing STRUCTURE output and implementing the Evanno method. Conservation Genetics Resources, 4(2), 359–361. 10.1007/s12686-011-9548-7

[eva13629-bib-0031] Easton, L. J. , Bishop, P. J. , & Whigham, P. A. (2020). Balancing act: Modelling sustainable release numbers for translocations. Animal Conservation, 23(4), 434–442. 10.1111/acv.12558

[eva13629-bib-0032] Evanno, G. , Regnaut, S. , & Goudet, J. (2005). Detecting the number of clusters of individuals using the software STRUCTURE: A simulation study. Molecular Ecology, 14(8), 2611–2620. 10.1111/j.1365-294X.2005.02553.x 15969739

[eva13629-bib-0033] Excoffier, L. , & Lischer, H. E. L. (2010). Arlequin suite ver 3.5: A new series of programs to perform population genetics analyses under Linux and Windows. Molecular Ecology Resources, 10(3), 564–567. 10.1111/j.1755-0998.2010.02847.x 21565059

[eva13629-bib-0034] Falconer, D. S. , & Mackay, T. F. C. (1996). Introduction to quantitative genetics (4th edition) chapter 3: Small populations: I changes of gene frequency under simplified conditions. Pearson Education Ltd.

[eva13629-bib-0035] Flesch, E. P. , Graves, T. A. , Thomson, J. M. , Proffitt, K. M. , White, P. J. , Stephenson, T. R. , & Garrott, R. A. (2020). Evaluating wildlife translocations using genomics: A bighorn sheep case study. Ecology and Evolution, 10(24), 13687–13704. 10.1002/ece3.6942 33391673 PMC7771163

[eva13629-bib-0036] Frankham, R. (2015). Genetic rescue of small inbred populations: Meta‐analysis reveals large and consistent benefits of gene flow. Molecular Ecology, 24(11), 2610–2618. 10.1111/mec.13139 25740414

[eva13629-bib-0037] Frankham, R. , Ballou, J. D. , Eldridge, M. D. B. , Lacy, R. C. , Ralls, K. , Dudash, M. R. , & Fenster, C. B. (2011). Predicting the probability of outbreeding depression. Conservation Biology, 25(3), 465–475. 10.1111/j.1523-1739.2011.01662.x 21486369

[eva13629-bib-0038] Frankham, R. , Ballou, J. D. , Ralls, K. , Eldridge, M. , Dudash, M. R. , Fenster, C. B. , Lacy, R. C. , & Sunnucks, P. (2019). A practical guide for genetic management of fragmented animal and plant populations. Oxford University Press.

[eva13629-bib-0039] Godefroid, S. , Piazza, C. , Rossi, G. , Buord, S. , Stevens, A. D. , Aguraiuja, R. , Cowell, C. , Weekley, C. W. , Vogg, G. , Iriondo, J. M. , Johnson, I. , Dixon, B. , Gordon, D. , Magnanon, S. , Valentin, B. , Bjureke, K. , Koopman, R. , Vicens, M. , Virevaire, M. , & Vanderborght, T. (2011). How successful are plant species reintroductions? Biological Conservation, 144(2), 672–682.

[eva13629-bib-0040] Gosselin, T. , Lamothe, M. , Devloo‐Delva, F. , & Grewe, P. (2020). Radiator: RADseq data exploration, manipulation and visualization using R . 10.5281/zenodo.3687060

[eva13629-bib-0041] Goudet, J. (2005). HIERFSTAT, a package for R to compute and test hierarchical F‐statistics. Molecular Ecology Notes, 5(1), 184–186. 10.1111/j.1471-8286.2004.00828.x

[eva13629-bib-0042] Halley, D. J. (2011). Sourcing Eurasian beaver *Castor fiber* stock for reintroductions in Great Britain and Western Europe. Mammal Review, 41(1), 40–53. 10.1111/j.1365-2907.2010.00167.x

[eva13629-bib-0043] Halley, D. J. , & Rosell, F. N. (2003). Population and distribution of European beavers (*Castor fiber*). Lutra, 46, 91–101.

[eva13629-bib-0044] Halley, D. J. , Saveljev, A. P. , & Rosell, F. (2021). Population and distribution of beavers *Castor fiber* and *Castor canadensis* in Eurasia. Mammal Review, 51(1), 1–24. 10.1111/mam.12216

[eva13629-bib-0045] Hamilton, A. , & Moran, D. (2015). Tayside beaver socio‐economic impact study . Scottish Natural Heritage Commissioned Report No. 805.

[eva13629-bib-0046] Hanghøj, K. , Moltke, I. , Andersen, P. A. , Manica, A. , & Korneliussen, T. S. (2019). Fast and accurate relatedness estimation from high‐throughput sequencing data in the presence of inbreeding. GigaScience, 8(5), giz034. 10.1093/gigascience/giz034 31042285 PMC6488770

[eva13629-bib-0047] Hartman, G. (1997). Notes on age at dispersal of beaver (*Castor fiber*) in an expanding population. Canadian Journal of Zoology, 75(6), 959–962. 10.1139/z97-116

[eva13629-bib-0048] Hoban, S. , Bruford, M. , D'Urban Jackson, J. , Lopes‐Fernandes, M. , Heuertz, M. , Hohenlohe, P. A. , Paz‐Vinas, I. , Sjögren‐Gulve, P. , Segelbacher, G. , Vernesi, C. , Aitken, S. , Bertola, L. D. , Bloomer, P. , Breed, M. , Rodríguez‐Correa, H. , Funk, W. C. , Grueber, C. E. , Hunter, M. E. , Jaffe, R. , … Laikre, L. (2020). Genetic diversity targets and indicators in the CBD post‐2020 Global Biodiversity Framework must be improved. Biological Conservation, 248, 108654. 10.1016/j.biocon.2020.108654

[eva13629-bib-0049] Hoban, S. , Bruford, M. W. , Funk, W. C. , Galbusera, P. , Griffith, M. P. , Grueber, C. E. , Heuertz, M. , Hunter, M. E. , Hvilsom, C. , Stroil, B. K. , Kershaw, F. , Khoury, C. K. , Laikre, L. , Lopes‐Fernandes, M. , MacDonald, A. J. , Mergeay, J. , Meek, M. , Mittan, C. , Mukassabi, T. A. , … Vernesi, C. (2021). Global commitments to conserving and monitoring genetic diversity are now necessary and feasible. Bioscience, 71(9), 964–976. 10.1093/biosci/biab054 34475806 PMC8407967

[eva13629-bib-0050] Hogg, C. J. , Grueber, C. E. , Pemberton, D. , Fox, S. , Lee, A.v. , Ivy, J. A. , & Belov, K. (2017). “Devil tools & tech”: A synergy of conservation research and management practice. Conservation Letters, 10(1), 133–138. 10.1111/conl.12221

[eva13629-bib-0051] Horn, S. , Prost, S. , Stiller, M. , Makowiecki, D. , Kuznetsova, T. , Benecke, N. , Pucher, E. , Hufthammer, A. K. , Schouwenburg, C. , Shapiro, B. , & Hofreiter, M. (2014). Ancient mitochondrial DNA and the genetic history of Eurasian beaver (*Castor fiber*) in Europe. Molecular Ecology, 23(7), 1717–1729. 10.1111/mec.12691 24795996

[eva13629-bib-0052] IUCN Species Survival Commission . (2013). Guidelines for reintroductions and other conservation translocations. IUCN Species Survival Commission.

[eva13629-bib-0053] IUCN/CPSG . (2022). Scotland's Beaver Strategy 2022–2045 . https://www.nature.scot/doc/scotlands‐beaver‐strategy‐2022‐2045

[eva13629-bib-0054] James, J. W. (1970). The founder effect and response to artificial selection. Genetics Research, 16(3), 241–250. 10.1017/S0016672300002500 5512250

[eva13629-bib-0055] Jamieson, I. G. , Wallis, G. P. , & Briskie, J. V. (2006). Inbreeding and endangered species management: Is New Zealand out of step with the rest of the world? Conservation Biology, 20(1), 38–47. 16909657 10.1111/j.1523-1739.2005.00282.x

[eva13629-bib-0056] Jimenez, J. A. , Hughes, K. A. , Alaks, G. , Graham, L. , & Lacy, R. C. (1994). An experimental study of inbreeding depression in a natural habitat. Science, 266(5183), 271–273. 10.1126/science.7939661 7939661

[eva13629-bib-0057] Jombart, T. (2008). adegenet: A R package for the multivariate analysis of genetic markers. Bioinformatics, 24(11), 1403–1405. 10.1093/bioinformatics/btn129 18397895

[eva13629-bib-0058] Jombart, T. , & Ahmed, I. (2011). adegenet 1.3‐1: New tools for the analysis of genome‐wide SNP data. Bioinformatics, 27(21), 3070–3071. 10.1093/bioinformatics/btr521 21926124 PMC3198581

[eva13629-bib-0059] Kardos, M. , Armstrong, E. E. , Fitzpatrick, S. W. , Hauser, S. , Hedrick, P. W. , Miller, J. M. , Tallmon, D. A. , & Funk, W. C. (2021). The crucial role of genome‐wide genetic variation in conservation. Proceedings of the National Academy of Sciences of the United States of America, 118(48), e2104642118. 10.1073/pnas.2104642118 34772759 PMC8640931

[eva13629-bib-0060] Kemp, L. , Norbury, G. , Groenewegen, R. , & Comer, S. (2015). The role of trials and experiments in fauna reintroduction programmes. In D. Armstrong , M. Hayward , D. Moro , & P. Seddon (Eds.), Advances in reintroduction biology of Australian and New Zealand Fauna (pp. 73–89). CSIRO Publishing.

[eva13629-bib-0061] Kitchener, A. C. , & Conroy, J. W. H. (1997). The history of the Eurasian beaver *Castor fiber* in Scotland. Mammal Review, 27(2), 95–108. 10.1111/j.1365-2907.1997.tb00374.x

[eva13629-bib-0062] Kitchener, A. C. , & Lynch, J. M. (2000). A morphometric comparison of the skulls of fossil British and extant European beavers, *Castor fiber* . Scottish Natural Heritage Review, 127, 1–37.

[eva13629-bib-0063] Kopelman, N. M. , Mayzel, J. , Jakobsson, M. , Rosenberg, N. A. , & Mayrose, I. (2015). Clumpak: A program for identifying clustering modes and packaging population structure inferences across K. Molecular Ecology Resources, 15(5), 1179–1191. 10.1111/1755-0998.12387 25684545 PMC4534335

[eva13629-bib-0064] Köster, J. , & Rahmann, S. (2012). Snakemake – A scalable bioinformatics workflow engine. Bioinformatics (Oxford, England), 28(19), 2520–2522. 10.1093/bioinformatics/bts480 22908215

[eva13629-bib-0065] Lacy, R. , & Horner, B. (1997). Effects of inbreeding on reproduction and sex ratio of *Rattus villosissimus* . Journal of Mammalogy, 78, 877–887. 10.2307/1382946

[eva13629-bib-0066] Lacy, R. C. , Alaks, G. , & Walsh, A. (1996). Hierarchical analysis of inbreeding depression in *Peromyscus polionotus* . Evolution, 50(6), 2187–2200. 10.2307/2410690 28565659

[eva13629-bib-0067] Lavrov, L. S. (1973). Karyotypes and taxonomy of modern beavers (Castor, Castoridae, Mammalia). Zoologicheskiĭ Zhurnal, 52, 734–742.

[eva13629-bib-0068] Leigh, J. W. , & Bryant, D. (2015). POPART: Full‐feature software for haplotype network construction. Methods in Ecology and Evolution, 6(9), 1110–1116. 10.1111/2041-210X.12410

[eva13629-bib-0069] Li, H. , & Durbin, R. (2009). Fast and accurate short read alignment with Burrows‐Wheeler transform. Bioinformatics, 25(14), 1754–1760. 10.1093/bioinformatics/btp324 19451168 PMC2705234

[eva13629-bib-0070] Li, H. , Handsaker, B. , Wysoker, A. , Fennell, T. , Ruan, J. , Homer, N. , Marth, G. , Abecasis, G. , & Durbin, R. (2009). The Sequence Alignment/Map format and SAMtools. Bioinformatics, 25(16), 2078–2079. 10.1093/bioinformatics/btp352 19505943 PMC2723002

[eva13629-bib-0071] Liddell, E. , Sunnucks, P. , & Cook, C. N. (2021). To mix or not to mix gene pools for threatened species management? Few studies use genetic data to examine the risks of both actions, but failing to do so leads disproportionately to recommendations for separate management. Biological Conservation, 256, 109072. 10.1016/j.biocon.2021.109072

[eva13629-bib-0072] Liu, J. , Mooney, H. , Hull, V. , Davis, S. J. , Gaskell, J. , Hertel, T. , Lubchenco, J. , Seto, K. C. , Gleick, P. , Kremen, C. , & Li, S. (2015). Systems integration for global sustainability. Science, 347(6225). 10.1126/science.1258832 25722418

[eva13629-bib-0073] Lok, S. , Paton, T. A. , Wang, Z. , Kaur, G. , Walker, S. , Yuen, R. K. C. , Sung, W. W. L. , Whitney, J. , Buchanan, J. A. , Trost, B. , Singh, N. , Apresto, B. , Chen, N. , Coole, M. , Dawson, T. J. , Ho, K. , Hu, Z. , Pullenayegum, S. , Samler, K. , … Scherer, S. W. (2017). De novo genome and transcriptome assembly of the Canadian beaver (*Castor canadensis*). G3: Genes, Genomes, Genetics, 7(2), 755–773. 10.1534/g3.116.038208 28087693 PMC5295618

[eva13629-bib-0074] Manichaikul, A. , Mychaleckyj, J. C. , Rich, S. S. , Daly, K. , Sale, M. , & Chen, W.‐M. (2010). Robust relationship inference in genome‐wide association studies. Bioinformatics, 26(22), 2867–2873. 10.1093/bioinformatics/btq559 20926424 PMC3025716

[eva13629-bib-0075] Manousaki, T. , Tsakogiannis, A. , Taggart, J. B. , Palaiokostas, C. , Tsaparis, D. , Lagnel, J. , Chatziplis, D. , Magoulas, A. , Papandroulakis, N. , Mylonas, C. C. , & Tsigenopoulos, C. S. (2015). Exploring a nonmodel teleost genome through RAD sequencing‐linkage mapping in common Pandora, *Pagellus erythrinus* and comparative genomic analysis. G3 (Bethesda, Md.), 6(3), 509–519. 10.1534/g3.115.023432 26715088 PMC4777114

[eva13629-bib-0076] Mayer, M. , Zedrosser, A. , & Rosell, F. (2017). When to leave: The timing of natal dispersal in a large, monogamous rodent, the Eurasian beaver. Animal Behaviour, 123, 375–382. 10.1016/j.anbehav.2016.11.020

[eva13629-bib-0077] McEwing, R. , Senn, H. , & Campbell‐Palmer, R. (2015). Genetic assessment of free‐living beavers in and around the River Tay catchment, east Scotland Scottish Natural Heritage Commissioned Report No. 682 .

[eva13629-bib-0078] Miller, K. A. , Nelson, N. J. , Smith, H. G. , & Moore, J. A. (2009). How do reproductive skew and founder group size affect genetic diversity in reintroduced populations? Molecular Ecology, 18(18), 3792–3802. 10.1111/j.1365-294X.2009.04315.x 19732338

[eva13629-bib-0079] Murphy, S. M. , Adams, J. R. , Waits, L. P. , & Cox, J. J. (2021). Evaluating otter reintroduction outcomes using genetic spatial capture–recapture modified for dendritic networks. Ecology and Evolution, 11(21), 15047–15061. 10.1002/ece3.8187 34765159 PMC8571598

[eva13629-bib-0080] Nolet, B. A. , & Rosell, F. (1998). Comeback of the beaver *Castor fiber*: An overview of old and new conservation problems. Biological Conservation, 83(2), 165–173. 10.1016/S0006-3207(97)00066-9

[eva13629-bib-0081] Norman, J. A. , & Christidis, L. (2021). A spatial genetic framework for koala translocations: Where to? Wildlife Research, 48(3), 193–201. 10.1071/WR20055

[eva13629-bib-0082] Ogden, R. , Chuven, J. , Gilbert, T. , Hosking, C. , Gharbi, K. , Craig, M. , Al Dhaheri, S. S. , & Senn, H. (2020). Benefits and pitfalls of captive conservation genetic management: Evaluating diversity in scimitar‐horned oryx to support reintroduction planning. Biological Conservation, 241, 108244. 10.1016/j.biocon.2019.108244

[eva13629-bib-0083] Olazcuaga, L. , Lincke, B. , DeLacey, S. , Durkee, L. F. , Melbourne, B. A. , & Hufbauer, R. A. (2023). Population demographic history and evolutionary rescue: Influence of a bottleneck event. Evolutionary Applications, 16(8), 1483–1495. 10.1111/eva.13581 37622091 PMC10445088

[eva13629-bib-0084] O'Leary, S. J. , Puritz, J. B. , Willis, S. C. , Hollenbeck, C. M. , & Portnoy, D. S. (2018). These aren't the loci you'e looking for: Principles of effective SNP filtering for molecular ecologists. Molecular Ecology, 27(16), 3193–3206. 10.1111/mec.14792 29987880

[eva13629-bib-0085] Olson, L. E. , Blumstein, D. T. , Pollinger, J. R. , & Wayne, R. K. (2012). No evidence of inbreeding avoidance despite demonstrated survival costs in a polygynous rodent. Molecular Ecology, 21(3), 562–571. 10.1111/j.1365-294X.2011.05389.x 22145620

[eva13629-bib-0086] Pacioni, C. , Rafferty, C. , Morley, K. , Stevenson, S. , Chapman, A. , Wickins, M. , Verney, T. , Deegan, G. , Trocini, S. , & Spencer, P. B. S. (2018). Augmenting the conservation value of rehabilitated wildlife by integrating genetics and population modeling in the post‐rehabilitation decision process. Current Zoology, 64(5), 593–601. 10.1093/cz/zox065 30323838 PMC6178788

[eva13629-bib-0087] Pacioni, C. , Trocini, S. , Wayne, A. F. , Rafferty, C. , & Page, M. (2020). Integrating population genetics in an adaptive management framework to inform management strategies. Biodiversity and Conservation, 29(3), 947–966. 10.1007/s10531-019-01920-7

[eva13629-bib-0088] Peterson, B. K. , Weber, J. N. , Kay, E. H. , Fisher, H. S. , & Hoekstra, H. E. (2012). Double digest RADseq: An inexpensive method for de novo SNP discovery and genotyping in model and non‐model species. PLoS One, 7(5), e37135. 10.1371/journal.pone.0037135 22675423 PMC3365034

[eva13629-bib-0089] Pierson, J. C. , Coates, D. J. , Oostermeijer, J. G. B. , Beissinger, S. R. , Bragg, J. G. , Sunnucks, P. , Schumaker, N. H. , & Young, A. G. (2016). Genetic factors in threatened species recovery plans on three continents. Frontiers in Ecology and the Environment, 14(8), 433–440. 10.1002/fee.1323

[eva13629-bib-0090] Pritchard, J. K. , Stephens, M. , & Donnelly, P. (2000). Inference of population structure using multilocus genotype data. Genetics, 155(2), 945–959. 10.1111/j.1471-8286.2007.01758.x 10835412 PMC1461096

[eva13629-bib-0091] Pritchard, J. K. , Wen, X. , & Falush, D. (2007). Documentation for structure software: Version 2.2 (pp. 1–36). University of Chicago.

[eva13629-bib-0092] Pucci, C. , Senserini, D. , Mazza, G. , & Mori, E. (2021). Reappearance of the Eurasian beaver *Castor fiber* L. in Tuscany (Central Italy): The success of *unauthorised* releases? Hystrix, the Italian Journal of Mammalogy, 32(2), 182–185. 10.4404/hystrix-00445-2021

[eva13629-bib-0093] Purcell, S. , Neale, B. , Todd‐Brown, K. , Thomas, L. , Ferreira, M. A. , Bender, D. , Maller, J. , Sklar, P. , de Bakker, P. I. , Daly, M. J. , & Sham, P. C. (2007). PLINK: A tool set for whole‐genome association and population‐based linkage analyses. American Journal of Human Genetics, 81(3), 559–575. 10.1086/519795 17701901 PMC1950838

[eva13629-bib-0094] Ralls, K. , Ballou, J. D. , Dudash, M. R. , Eldridge, M. D. B. , Fenster, C. B. , Lacy, R. C. , Sunnucks, P. , & Frankham, R. (2018). Call for a paradigm shift in the genetic management of fragmented populations. Conservation Letters, 11(2), e12412. 10.1111/conl.12412

[eva13629-bib-0095] Ramstad, K. M. , Colbourne, R. M. , Robertson, H. A. , Allendorf, F. W. , & Daugherty, C. H. (2013). Genetic consequences of a century of protection: Serial founder events and survival of the little spotted kiwi (*Apteryx owenii*). Proceedings of the Royal Society B, 280, 20130576. 10.1098/rspb.2013.0576 23677342 PMC3673049

[eva13629-bib-0096] Robertson, A. , & Waddington, C. H. (1997). A theory of limits in artificial selection. Proceedings of the Royal Society of London. Series B. Biological Sciences, 153(951), 234–249. 10.1098/rspb.1960.0099

[eva13629-bib-0097] Robledo‐Ruiz, D. A. , Pavlova, A. , Clarke, R. H. , Magrath, M. J. L. , Quin, B. , Harrisson, K. A. , Gan, H. M. , Low, G. W. , & Sunnucks, P. (2022). A novel framework for evaluating in situ breeding management strategies in endangered populations. Molecular Ecology Resources, 22(1), 239–253. 10.1111/1755-0998.13476 34288508

[eva13629-bib-0098] Rochette, N. C. , Rivera‐Colón, A. G. , & Catchen, J. M. (2019). Stacks 2: Analytical methods for paired‐end sequencing improve RADseq‐based population genomics. Molecular Ecology, 28(21), 4737–4754. 10.1111/mec.15253 31550391

[eva13629-bib-0099] Ross‐Gillespie, A. , O'Riain, M. J. , & Keller, L. F. (2007). Viral epizootic reveals inbreeding depression in a habitually inbreeding mammal. Evolution, 61(9), 2268–2273. 10.1111/j.1558-5646.2007.00177.x 17767596 PMC7202238

[eva13629-bib-0100] Scottish Natural Heritage . (2015). Beavers in Scotland: A report to the Scottish Government .

[eva13629-bib-0101] Senn, H. V. , Ghazali, M. , Kaden, J. , Barclay, D. , Harrower, B. , Campbell, R. D. , Macdonald, D. W. , & Kitchener, A. C. (2019). Distinguishing the victim from the threat: SNP‐based methods reveal the extent of introgressive hybridization between wildcats and domestic cats in Scotland and inform future in situ and ex situ management options for species restoration. Evolutionary Applications, 12(3), 399–414. 10.1111/eva.12720 30828363 PMC6383845

[eva13629-bib-0102] Senn, H. , Ogden, R. , Frosch, C. , Syrůčková, A. , Campbell‐Palmer, R. , Munclinger, P. , Durka, W. , Kraus, R. H. S. , Saveljev, A. P. , Nowak, C. , Stubbe, A. , Stubbe, M. , Michaux, J. , Lavrov, V. , Samiya, R. , Ulevicius, A. , & Rosell, F. (2014). Nuclear and mitochondrial genetic structure in the Eurasian beaver (*Castor fiber*) – Implications for future reintroductions. Evolutionary Applications, 7(6), 645–662. 10.1111/eva.12162 25067948 PMC4105916

[eva13629-bib-0103] Shafer, A. B. A. , Peart, C. R. , Tusso, S. , Maayan, I. , Brelsford, A. , Wheat, C. W. , & Wolf, J. B. W. (2016). Bioinformatic processing of RAD‐seq data dramatically impacts downstream population genetic inference. Methods in Ecology and Evolution, 8, 907–917. 10.1111/2041-210X.12700

[eva13629-bib-0104] Shapcott, A. , Olsen, M. , & Lamont, R. W. (2009). The importance of genetic considerations for planning translocations of the rare coastal heath species *Boronia rivularis* (Rutaceae) in Queensland. Ecological Restoration, 27(1), 47–57.

[eva13629-bib-0105] Stringer, A. P. , & Gaywood, M. J. (2016). The impacts of beavers *Castor* spp. on biodiversity and the ecological basis for their reintroduction to Scotland, UK. Mammal Review, 46(4), 270–283. 10.1111/mam.12068

[eva13629-bib-0106] Syrůčková, A. , Saveljev, A. P. , Frosch, C. , Durka, W. , Savelyev, A. A. , & Munclinger, P. (2015). Genetic relationships within colonies suggest genetic monogamy in the Eurasian beaver (*Castor fiber*). Mammal Research, 60(2), 139–147. 10.1007/s13364-015-0219-z

[eva13629-bib-0107] Tallmon, D. A. , Luikart, G. , & Waples, R. S. (2004). The alluring simplicity and complex reality of genetic rescue. Trends in Ecology & Evolution, 19(9), 489–496. 10.1016/j.tree.2004.07.003 16701312

[eva13629-bib-0108] Taylor, H. R. , Colbourne, R. M. , Robertson, H. A. , Nelson, N. J. , Allendorf, F. W. , & Ramstad, K. M. (2017). Cryptic inbreeding depression in a growing population of a long‐lived species. Molecular Ecology, 26, 799–813. 10.1111/mec.13977 28093817

[eva13629-bib-0109] Taylor, H. R. , Dussex, N. , & van Heezik, Y. (2017). Bridging the conservation genetics gap by identifying barriers to implementation for conservation practitioners. Global Ecology and Conservation, 10, 231–242. 10.1016/j.gecco.2017.04.001

[eva13629-bib-0110] Teixeira, J. C. , & Huber, C. D. (2021). The inflated significance of neutral genetic diversity in conservation genetics. Proceedings of the National Academy of Sciences of the United States of America, 118(10), e2015096118. 10.1073/pnas.2015096118 33608481 PMC7958437

[eva13629-bib-0111] Thavornkanlapachai, R. , Mills, H. R. , Ottewell, K. , Dunlop, J. , Sims, C. , Morris, K. , Donaldson, F. , & Kennington, W. J. (2019). Mixing genetically and morphologically distinct populations in translocations: Asymmetrical introgression in A newly established population of the Boodie (*Bettongia lesueur*). Genes, 10(9), 729. 10.3390/genes10090729 31546973 PMC6770996

[eva13629-bib-0112] The Conference of the Parties to the Convention on Biological Diversity . (2022). Kunming‐Montreal Global biodiversity framework: Draft decision submitted by the President .

[eva13629-bib-0113] Thornton, T. , Tang, H. , Hoffmann, T. J. , Ochs‐Balcom, H. M. , Caan, B. J. , & Risch, N. (2012). Estimating kinship in admixed populations. The American Journal of Human Genetics, 91(1), 122–138. 10.1016/j.ajhg.2012.05.024 22748210 PMC3397261

[eva13629-bib-0114] Tracy, L. N. , & Jamieson, I. G. (2011). Preserving genetic diversity in threatened species reintroductions: How many individuals should be released? Animal Conservation, 14, 439–446.

[eva13629-bib-0115] Wahlund, S. (1928). Zusammensetzung von populationen und korrelationrscheinungen vom standpunkt der vererbungslehre aus betrachtet. Hereditas, 11(1), 65–106. 10.1111/j.1601-5223.1928.tb02483.x

[eva13629-bib-0116] Waples, R. K. , Albrechtsen, A. , & Moltke, I. (2019). Allele frequency‐free inference of close familial relationships from genotypes or low‐depth sequencing data. Molecular Ecology, 28(1), 35–48. 10.1111/mec.14954 30462358 PMC6850436

[eva13629-bib-0117] Weeks, A. R. , Moro, D. , Thavornkanlapachai, R. , Taylor, H. R. , White, N. E. , Weiser, E. L. , & Heinze, D. (2015). Conserving and enhancing genetic diversity in translocation programmes. In D. P. Armstrong , M. W. Hayward , D. Moro , & P. J. Seddon (Eds.), Advances in reintroduction biology of Australian and New Zealand Fauna (pp. 127–139). CSIRO Publishing.

[eva13629-bib-0118] Weiser, E. L. , Grueber, C. E. , & Jamieson, I. G. (2013). Simulating retention of rare alleles in small populations to assess management options for species with different life histories. Conservation Biology, 27(2), 335–344. 10.1111/cobi.12011 23330669

[eva13629-bib-0119] White, D. J. , Ottewell, K. , Spencer, P. B. S. , Smith, M. , Short, J. , Sims, C. , & Mitchell, N. J. (2020). Genetic consequences of multiple translocations of the banded hare‐wallaby in Western Australia. Diversity, 12(12), 448. 10.3390/d12120448

[eva13629-bib-0120] White, L. C. , Thomson, V. A. , West, R. , Ruykys, L. , Ottewell, K. , Kanowski, J. , Moseby, K. E. , Byrne, M. , Donnellan, S. C. , Copley, P. , & Austin, J. J. (2020). Genetic monitoring of the greater stick‐nest rat meta‐population for strategic supplementation planning. Conservation Genetics, 21(5), 941–956. 10.1007/s10592-020-01299-x

[eva13629-bib-0121] Wigginton, J. E. , Cutler, D. J. , & Abecasis, G. R. (2005). A note on exact tests of Hardy‐Weinberg equilibrium. American Journal of Human Genetics, 76(5), 887–893. 10.1086/429864 15789306 PMC1199378

[eva13629-bib-0122] Willi, Y. , Kristensen, T. N. , Sgrò, C. M. , Weeks, A. R. , Ørsted, M. , & Hoffmann, A. A. (2022). Conservation genetics as a management tool: The five best‐supported paradigms to assist the management of threatened species. Proceedings of the National Academy of Sciences of the United States of America, 119(1), e2105076119. 10.1073/pnas.2105076119 34930821 PMC8740573

[eva13629-bib-0123] Wilsson, L. (1971). *Observations and experiments on the ethology of the European beaver (*Castor fiber *L.): A study in the development of phylogenetically adapted behaviour in a highly specialized mammal* [PhD thesis]. Stockholm University.

[eva13629-bib-0124] Wright, S. (1931). Evolution in Mendelian populations. Genetics, 16(2), 97–159. 17246615 10.1093/genetics/16.2.97 PMC1201091

