# Supplementary material for: The genetic legacy of the first successful reintroduction of a mammal to Britain: Founder events and attempted genetic rescue in Scotland's beaver population
Source: Evol Appl. 2023 Dec 28;17(2):e13629. doi: 10.1111/eva.13629 (PMC10853653; doi:10.1111/eva.13629)
Supplement: Supplementary file 1 — File S1 [file EVA-17-e13629-s002.docx]

**Supplementary Information 1**

***De novo* SNP calling**

Previous studies have shown that SNP discovery using a reference genome of a closely related species to that of the focal species is reliable and relevant, particularly within a conservation context (Galla et al. 2019; Samaha et al. 2021). To confirm that this was the case when using the genome of North American beaver (*Castor canadensis*) as a reference for SNP calling within the Eurasian beaver (*Castor fiber*), we repeated all our analyses via de novo SNP calling within STACKS v2.52. We performed parameterisation tests following Paris et al (2017) and Rochette & Catchen (2017) for a subset of 22 individuals. This resulted in the selecting the parameters: the number of mismatches allowed between stacks within individuals (-M) and the number of mismatches allowed between stacks between individuals (-n) were set to two, and the minimum depth of coverage was set to five. SNP calling was carried out for all individuals with more than 250,000 raw reads using the *denovo_map.pl* pipeline. SNP filtering and population genetic analyses were carried out as described for reference-aligned SNP calling (see Methods). After SNP-filtering, we identified a total of 2190 SNPs in 104 individuals. Genetic diversity parameters (Table S2) were found to be highly concordant with those estimated from reference-aligned SNPs (Table 1, main manuscript).

Table S1: Sample information for the 104 individuals used in the analyses for this study. The group each sample belongs to corresponds to the groupings described in the main methods section titled “Population genetic analyses”. Note that the “Knapdale current” grouping contains some individuals that are also present in the “Knapdale trial” and “reinforcement” groups.

| **Sample** | **Sample type** | **Group 1** | **Group 2 (where relevant)** | **Was sample sequenced at mtDNA?** | **mtDNA haplotype** | **mtDNA reference** | **Was sample genotyped by ddRAD?** | **ddRAD ID*** | **ddRAD data reference** | **Biosample accession** |
| --- | --- | --- | --- | --- | --- | --- | --- | --- | --- | --- |
| BEV001 | tissue | Bavaria | NA | Yes | jf7 | Senn et al 2014 | Yes | BEV001_49 | This paper | SAMN29862098 |
| BEV002 | tissue | Bavaria | NA | Yes | jf7 | Senn et al 2014 | Yes | BEV002_49 | This paper | SAMN29862099 |
| BEV003 | tissue | Bavaria | NA | Yes | ga1 | Senn et al 2014 | Yes | BEV003_49 | This paper | SAMN29862100 |
| BEV005 | tissue | Bavaria | NA | Yes | ga1 | Senn et al 2014 | Yes | BEV005_49 | This paper | SAMN29862101 |
| BEV006 | tissue | Bavaria | NA | Yes | jf7 | Senn et al 2014 | Yes | BEV006_49 | This paper | SAMN29862102 |
| BEV007 | tissue | Bavaria | NA | Yes | jf7 | Senn et al 2014 | Yes | BEV007_49 | This paper | SAMN29862103 |
| BEV008 | tissue | Bavaria | NA | Yes | jf7 | Senn et al 2014 | Yes | BEV008_49 | This paper | SAMN29862104 |
| BEV009 | tissue | Bavaria | NA | Yes | jf7 | Senn et al 2014 | Yes | BEV009_49 | This paper | SAMN29862105 |
| BEV040 | tissue | Bavaria | NA | Yes | jf7 | Senn et al 2014 | Yes | BEV040_49 | This paper | SAMN29862116 |
| BEV041 | tissue | Bavaria | NA | Yes | jf7 | Senn et al 2014 | Yes | BEV041_49 | This paper | SAMN29862117 |
| BEV043 | tissue | Bavaria | NA | Yes | jf7 | Senn et al 2014 | Yes | BEV043_49 | This paper | SAMN29862118 |
| BEV044 | tissue | Bavaria | NA | Yes | jf7 | Senn et al 2014 | Yes | BEV044_49 | This paper | SAMN29862119 |
| BEV045 | tissue | Bavaria | NA | Yes | jf7 | Senn et al 2014 | Yes | BEV045_49 | This paper | SAMN29862120 |
| BEV046 | tissue | Bavaria | NA | Yes | ga1 | Senn et al 2014 | Yes | BEV046_49 | This paper | SAMN29862121 |
| BEV047 | tissue | Bavaria | NA | Yes | ga1 | Senn et al 2014 | Yes | BEV047_49 | This paper | SAMN29862122 |
| BEV048 | tissue | Bavaria | NA | Yes | jf7 | Senn et al 2014 | Yes | BEV048_49 | This paper | SAMN29862123 |
| BEV049 | tissue | Bavaria | NA | Yes | ga1 | Senn et al 2014 | Yes | BEV049_49 | This paper | SAMN29862124 |
| BEV607 | blood | Knapdale current | Knapdale BORN | Yes | fi1 | This paper | Yes | BEV607_47 | This paper | SAMN29862155 |
| BEV779 | blood | Knapdale current | Knapdale BORN | Yes | fi1 | This paper | Yes | BEV779_47 | This paper | SAMN29862191 |
| BEV780 | blood | Knapdale current | Reinforcement | Yes | jf7 | This paper | Yes | BEV780_47 | This paper | SAMN29862192 |
| BEV784 | blood | Knapdale current | Knapdale BORN | Yes | fi1 | This paper | Yes | BEV784_47 | This paper | SAMN29862194 |
| BEV785 | blood | Knapdale current | Knapdale BORN | Yes | fi1 | This paper | Yes | BEV785_47 | This paper | SAMN29862195 |
| BEV787 | blood | Knapdale current | Knapdale BORN | Yes | fi1 | This paper | Yes | BEV787_47 | This paper | SAMN29862197 |
| BEV788 | blood | Knapdale current | Knapdale BORN | Yes | ga1 | This paper | Yes | BEV788_47 | This paper | SAMN29862198 |
| BEV306 | blood | Knapdale trial | Knapdale current | Yes | fi1 | This paper | Yes | BEV306_47 | This paper | SAMN29862125 |
| BEV307 | blood | Knapdale trial | NA | Yes | fi1 | This paper | Yes | BEV307_47 | This paper | SAMN29862126 |
| BEV308 | blood | Knapdale trial | Knapdale current | Yes | fi1 | This paper | Yes | BEV308_47 | This paper | SAMN29862127 |
| BEV309 | blood | Knapdale trial | Knapdale current | Yes | fi1 | This paper | Yes | BEV309_47 | This paper | SAMN29862128 |
| BEV310 | blood | Knapdale trial | Knapdale current | Yes | fi1 | This paper | Yes | BEV310_47 | This paper | SAMN29862129 |
| BEV314 | blood | Knapdale trial | Knapdale current | Yes | fi1 | This paper | Yes | BEV314_47 | This paper | SAMN29862131 |
| BEV400 | blood | Knapdale trial | Knapdale current | Yes | fi1 | This paper | Yes | BEV400_47 | This paper | SAMN29862150 |
| BEV401 | blood | Knapdale trial | NA | Yes | fi1 | This paper | Yes | BEV401_47 | This paper | SAMN29862151 |
| BEV402 | blood | Knapdale trial | NA | Yes | fi1 | This paper | Yes | BEV402_47 | This paper | SAMN29862152 |
| BEV608 | blood | Knapdale trial | NA | Yes | fi1 | This paper | Yes | BEV608_47 | This paper | SAMN29862156 |
| BEV786 | blood | Knapdale trial | Knapdale current | Yes | fi1 | This paper | Yes | BEV786_47 | This paper | SAMN29862196 |
| BEV013 | blood | Norway | NA | Yes | fi1 | Senn et al 2014 | Yes | BEV013_47 | This paper | SAMN29862106 |
| BEV014 | blood | Norway | NA | Yes | fi1 | Senn et al 2014 | Yes | BEV014_47 | This paper | SAMN29862107 |
| BEV015 | blood | Norway | NA | Yes | fi1 | Senn et al 2014 | Yes | BEV015_49 | This paper | SAMN29862108 |
| BEV017 | blood | Norway | NA | Yes | fi1 | Senn et al 2014 | Yes | BEV017_49 | This paper | SAMN29862109 |
| BEV018 | blood | Norway | NA | Yes | fi1 | Senn et al 2014 | Yes | BEV018_47 | This paper | SAMN29862110 |
| BEV019 | blood | Norway | NA | Yes | fi1 | Senn et al 2014 | Yes | BEV019.1_49 | This paper | SAMN29862111 |
| BEV020 | blood | Norway | NA | Yes | fi1 | Senn et al 2014 | Yes | BEV020_49 | This paper | SAMN29862112 |
| BEV022 | blood | Norway | NA | Yes | fi1 | Senn et al 2014 | Yes | BEV022_47 | This paper | SAMN29862113 |
| BEV034 | blood | Norway | NA | Yes | fi1 | Senn et al 2014 | Yes | BEV034_47 | This paper | SAMN29862114 |
| BEV036 | tissue | Norway | NA | Yes | fi1 | Senn et al 2014 | Yes | BEV036_47 | This paper | SAMN29862115 |
| BEV313 | blood | Norway | NA | Yes | fi1 | Senn et al 2014 | Yes | BEV313_47 | This paper | SAMN29862130 |
| BEV348 | tissue | Norway | NA | Yes | fi1 | Senn et al 2014 | Yes | BEV348_47 | This paper | SAMN29862132 |
| BEV349 | tissue | Norway | NA | Yes | fi1 | Senn et al 2014 | Yes | BEV349_47 | This paper | SAMN29862133 |
| BEV350 | tissue | Norway | NA | Yes | fi1 | Senn et al 2014 | Yes | BEV350_49 | This paper | SAMN29862134 |
| BEV351 | tissue | Norway | NA | Yes | fi1 | Senn et al 2014 | Yes | BEV351_49 | This paper | SAMN29862135 |
| BEV353 | tissue | Norway | NA | Yes | fi1 | Senn et al 2014 | Yes | BEV353_47 | This paper | SAMN29862136 |
| BEV355 | tissue | Norway | NA | Yes | fi1 | Senn et al 2014 | Yes | BEV355_49 | This paper | SAMN29862137 |
| BEV357 | tissue | Norway | NA | No | NA | NA | Yes | BEV357_47 | This paper | SAMN29862138 |
| BEV362 | tissue | Norway | NA | Yes | fi1 | Senn et al 2014 | Yes | BEV362_49 | This paper | SAMN29862139 |
| BEV363 | tissue | Norway | NA | Yes | fi1 | Senn et al 2014 | Yes | BEV363_49 | This paper | SAMN29862140 |
| BEV365 | tissue | Norway | NA | Yes | fi1 | Senn et al 2014 | Yes | BEV365_49 | This paper | SAMN29862141 |
| BEV366 | tissue | Norway | NA | Yes | fi1 | Senn et al 2014 | Yes | BEV366_49 | This paper | SAMN29862142 |
| BEV367 | tissue | Norway | NA | Yes | fi1 | Senn et al 2014 | Yes | BEV367_49 | This paper | SAMN29862143 |
| BEV368 | tissue | Norway | NA | No | NA | NA | Yes | BEV368_49 | This paper | SAMN29862144 |
| BEV377 | tissue | Norway | NA | Yes | fi1 | Senn et al 2014 | Yes | BEV377_49 | This paper | SAMN29862145 |
| BEV380 | tissue | Norway | NA | No | NA | NA | Yes | BEV380_47 | This paper | SAMN29862146 |
| BEV381 | tissue | Norway | NA | No | NA | NA | Yes | BEV381b_49 | This paper | SAMN29862147 |
| BEV403 | blood | Norway | NA | Yes | fi1 | Senn et al 2014 | Yes | BEV403_47 | This paper | SAMN29862153 |
| BEV657 | hair | Norway | NA | Yes | fi1 | Senn et al 2014 | Yes | BEV657_47 | This paper | SAMN29862165 |
| BEV754 | blood | Reinforcement | Knapdale current | Yes | jf7 | This paper | Yes | BEV754_47 | This paper | SAMN29862177 |
| BEV755 | blood | Reinforcement | Knapdale current | Yes | jf7 | This paper | Yes | BEV755_47 | This paper | SAMN29862178 |
| BEV756 | blood | Reinforcement | Knapdale current | Yes | jf7 | This paper | Yes | BEV756_47 | This paper | SAMN29862179 |
| BEV761 | blood | Reinforcement | Knapdale current | Yes | jf7 | This paper | Yes | BEV761_47 | This paper | SAMN29862180 |
| BEV762 | blood | Reinforcement | NA | Yes | ga1 | This paper | Yes | BEV762_47 | This paper | SAMN29862181 |
| BEV765 | blood | Reinforcement | Knapdale current | Yes | ga1 | This paper | Yes | BEV765_47 | This paper | SAMN29862182 |
| BEV766 | blood | Reinforcement | NA | Yes | jf7 | This paper | Yes | BEV766_47 | This paper | SAMN29862183 |
| BEV767 | blood | Reinforcement | Knapdale current | Yes | ga1 | This paper | Yes | BEV767_47 | This paper | SAMN29862184 |
| BEV768 | blood | Reinforcement | Knapdale current | Yes | jf7 | This paper | Yes | BEV768b_47 | This paper | SAMN29862185 |
| BEV769 | blood | Reinforcement | Knapdale current | Yes | ga1 | This paper | Yes | BEV769_47 | This paper | SAMN29862186 |
| BEV770 | blood | Reinforcement | Knapdale current | Yes | jf7 | This paper | Yes | BEV770_47 | This paper | SAMN29862187 |
| BEV773 | blood | Reinforcement | Knapdale current | Yes | jf7 | This paper | Yes | BEV773_47 | This paper | SAMN29862188 |
| BEV777 | blood | Reinforcement | Knapdale current | Yes | jf7 | This paper | Yes | BEV777_47 | This paper | SAMN29862189 |
| BEV778 | blood | Reinforcement | Knapdale current | Yes | jf7 | This paper | Yes | BEV778_47 | This paper | SAMN29862190 |
| BEV781 | blood | Reinforcement | Knapdale current | Yes | jf7 | This paper | Yes | BEV781_47 | This paper | SAMN29862193 |
| BEV790 | blood | Reinforcement | Knapdale current | Yes | jf7 | This paper | Yes | BEV790_47 | This paper | SAMN29862199 |
| BEV791 | blood | Reinforcement | NA | Yes | jf7 | This paper | Yes | BEV791_47 | This paper | SAMN29862200 |
| BEV792 | blood | Reinforcement | Knapdale current | Yes | jf7 | This paper | Yes | BEV792_47 | This paper | SAMN29862201 |
| BEV384 | tissue | Tayside | NA | Yes | jf7 | This paper | Yes | BEV384_47 | This paper | SAMN29862148 |
| BEV385 | tissue | Tayside | NA | No | NA | NA | Yes | BEV385b_49 | This paper | SAMN29862149 |
| BEV602 | tissue | Tayside | NA | Yes | fi1 | This paper | Yes | BEV602_47 | This paper | SAMN29862154 |
| BEV609 | blood | Tayside | NA | Yes | jf7 | This paper | Yes | BEV609_47 | This paper | SAMN29862157 |
| BEV610 | blood | Tayside | NA | Yes | jf7 | This paper | Yes | BEV610_47 | This paper | SAMN29862158 |
| BEV611 | blood | Tayside | NA | Yes | jf7 | This paper | Yes | BEV611_47 | This paper | SAMN29862159 |
| BEV615 | blood | Tayside | NA | Yes | jf7 | This paper | Yes | BEV615_47 | This paper | SAMN29862160 |
| BEV617 | blood | Tayside | NA | Yes | jf7 | This paper | Yes | BEV617_47 | This paper | SAMN29862161 |
| BEV622 | blood | Tayside | NA | Yes | jf7 | This paper | Yes | BEV622_47 | This paper | SAMN29862162 |
| BEV623 | blood | Tayside | NA | Yes | jf7 | This paper | Yes | BEV623_47 | This paper | SAMN29862163 |
| BEV627 | blood | Tayside | NA | Yes | jf7 | This paper | Yes | BEV627_47 | This paper | SAMN29862164 |
| BEV726 | tissue | Tayside | NA | Yes | jf7 | This paper | Yes | BEV726_47 | This paper | SAMN29862166 |
| BEV732 | tissue | Tayside | NA | Yes | ga1 | This paper | Yes | BEV732_47 | This paper | SAMN29862167 |
| BEV733 | tissue | Tayside | NA | Yes | jf7 | This paper | Yes | BEV733_47 | This paper | SAMN29862168 |
| BEV734 | tissue | Tayside | NA | Yes | ga1 | This paper | Yes | BEV734_47 | This paper | SAMN29862169 |
| BEV735 | tissue | Tayside | NA | Yes | fi1 | This paper | Yes | BEV735_47 | This paper | SAMN29862170 |
| BEV737 | tissue | Tayside | NA | Yes | jf7 | This paper | Yes | BEV737_47 | This paper | SAMN29862171 |
| BEV738 | tissue | Tayside | NA | Yes | jf7 | This paper | Yes | BEV738_47 | This paper | SAMN29862172 |
| BEV739 | tissue | Tayside | NA | Yes | jf7 | This paper | Yes | BEV739_47 | This paper | SAMN29862173 |
| BEV741 | tissue | Tayside | NA | Yes | jf7 | This paper | Yes | BEV741_47 | This paper | SAMN29862174 |
| BEV742 | tissue | Tayside | NA | Yes | jf7 | This paper | Yes | BEV742_47 | This paper | SAMN29862175 |
| BEV753 | blood | Tayside | NA | No | NA | NA | Yes | BEV753A_47 | This paper | SAMN29862176 |

Notes:

* ddRAD ID corresponds to the sample ID within the plink genotypes data file (.ped)

NA indicates not applicable

The raw ddRAD data is available at the NCBI SRA (https://www.ncbi.nlm.nih.gov/sra) under bioproject accession PRJNA860774.

Table S2: Genetic diversity summary for Scotland’s beavers, comparing them with their relative source populations, based on 104 individuals genotyped at 2190 SNPs identified through *de novo* SNP calling.

|  | **N** | **H_o_** | **H_s_** | **Ar (n=11)** | **F_IS_** | **Fixed loci (Prop.)** | **KING-robust** |
| --- | --- | --- | --- | --- | --- | --- | --- |
| **Norway reference** | 29 | 0.048 | 0.047 | 1.115 | 0.015 | 1898 | 0.013 |
| **(CI 95%)** |  | (0.042 - 0.054) | (0.042 - 0.053) | (1.102 - 1.128) | (-0.025 - 0.056) |  | (0.002 - 0.024) |
| **Knapdale trial** | 11 | 0.053 | 0.046 | 1.111 | -0.106 | 1924 | 0.151 |
| **(CI 95%)** |  | (0.046 - 0.060) | (0.041 - 0.052) | (1.100 - 1.124) | (-0.152 - -0.061) |  | (0.127 - 0.176) |
| **Knapdale current** | 29* | 0.200 | 0.305 | 1.790 | 0.247 | 86 | 0.151 |
| **(CI 95%)** |  | (0.194 - 0.206) | (0.298 - 0.312) | (1.779 - 1.802) | (0.232 – 0.263) |  | (0.127 - 0.176) |
| **Reinforcement** | 19 | 0.296 | 0.304 | 1.773 | 0.028 | 234 | -0.019 |
| **(CI 95%)** |  | (0.287 - 0.304) | (0.296 - 0.311) | (1.759 - 1.787) | (0.016 - 0.040) |  | (-0.039 -0.001) |
| **Tayside reference** | 22 | 0.290 | 0.302 | 1.775 | 0.046 | 182 | -0.024 |
| **(CI 95%)** |  | (0.281 - 0.298) | (0.294 - 0.310) | (1.772 - 1.788) | (0.035 - 0.058) |  | (-0.037 - -0.010) |
| **Bavaria reference** | 17 | 0.254 | 0.277 | 1.685 | 0.074 | 466 | -0.080 |
| **(CI 95%)** |  | (0.245 - 0.262) | (0.269 - 0.285) | (1.669 - 1.702) | (0.061 - 0.088) |  | (-0.097 - 0.064) |

H_O_ ­­– observed heterozygosity; H_E_ – expected heterozygosity; Ar – Allelic richness; F_IS_ – fixation index; CI – confidence interval

* Note that this category includes 6 individuals born in Knapdale and 23 individuals that are also included in other categories (7 in Knapdale trial, 16 in Reinforcement)

Table S3: Currently known mitochondrial haplotypes for Eurasian beavers with relevant accession numbers and original source publications for characterisation

| **Haplotype name** | **Accession number** | **Source reference** |
| --- | --- | --- |
| nh2 | KJ670496 | Senn et al 2014 |
| nh3 | KJ670497 | Senn et al 2014 |
| nh4 | KJ670498 | Senn et al 2014 |
| nh5 | KJ670499 | Senn et al 2014 |
| jf7 | JF264887 | Horn et al 2010 |
| al1 | DQ088700 | Durka et al 2005 |
| ga1 | DQ088703 | Durka et al 2005 |
| fi1 | DQ088702 | Durka et al 2005 |
| tu1 | AY623637 | Ducroz et al 2005 |
| tu2 | AY623638 | Ducroz et al 2005 |
| tu3 | AY623639 | Ducroz et al 2005 |
| tu4 | AY623640 | Ducroz et al 2005 |
| bi1 | AY623632 | Ducroz et al 2005 |
| po1 | AY623635 | Ducroz et al 2005 |
| po2 | AY623636 | Ducroz et al 2005 |
| in3 | AY623643 | Ducroz et al 2005 |
| in2 | AY623642 | Ducroz et al 2005 |

Ducroz, J.F., Stubbe, M., Saveljev, A.P., Heidecke, D., Samjaa, R., Ulevičius, A., Stubbe, A. and Durka, W., 2005. Genetic variation and population structure of the Eurasian beaver Castor fiber in Eastern Europe and Asia. *Journal of Mammalogy*, *86*(6), pp.1059-1067.

Durka, W., Babik, W., DUCROZ, J.F., Heidecke, D., Rosell, F., Samjaa, R., P. SAVELJEV, A.L.E.X.A.N.D.E.R., Stubbe, A., Ulevičius, A. and Stubbe, M., 2005. Mitochondrial phylogeography of the Eurasian beaver Castor fiber L. *Molecular ecology*, *14*(12), pp.3843-3856.

Horn, S., J. Teubner, J. Teubner, and D. Heidecke 2010. Mitochondrial DNA of beavers (Castor) in Germany. Artenschutzreport 26:72–78.

Senn, H., Ogden, R., Frosch, C., Syrůčková, A., Campbell-Palmer, R., Munclinger, P., Durka, W., Kraus, R. H. S., Saveljev, A. P., Nowak, C., Stubbe, A., Stubbe, M., Michaux, J., Lavrov, V., Samiya, R., Ulevicius, A., & Rosell, F. (2014). Nuclear and mitochondrial genetic structure in the Eurasian beaver (Castor fiber) – implications for future reintroductions. *Evolutionary Applications*, *7*(6), 645–662


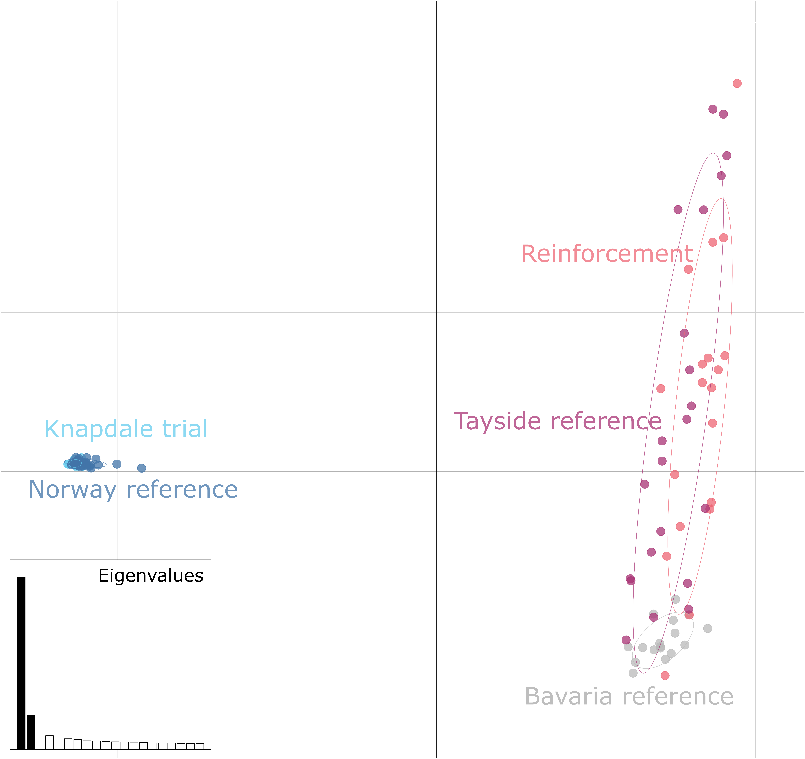


Figure S1. Principal component analysis of 104 individuals genotyped at 2190 SNPs identified *de novo.*


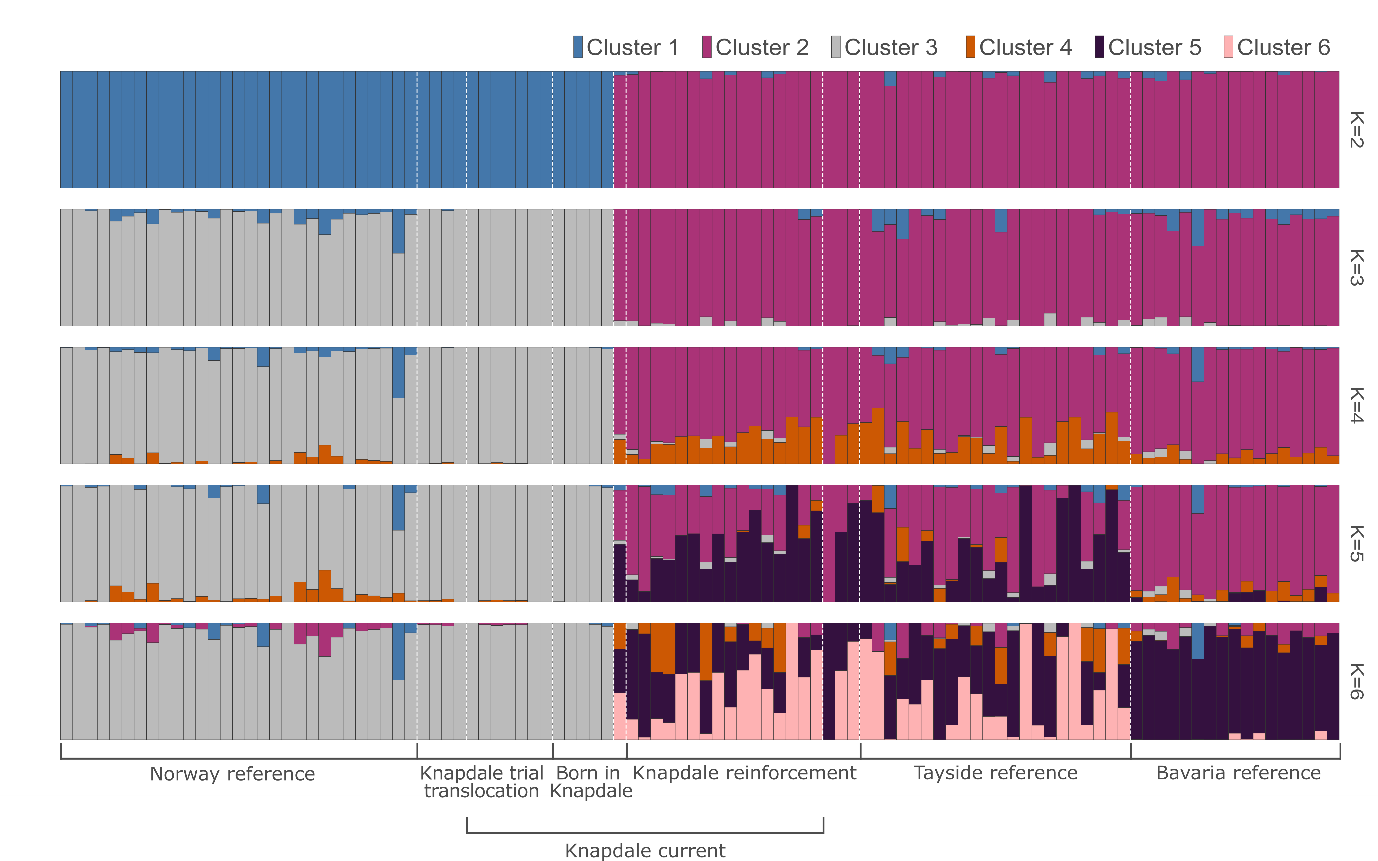


Figure S2. Visualisation of STRUCTURE results for 104 individuals genotyped at 2190 SNPs identified *de novo.*


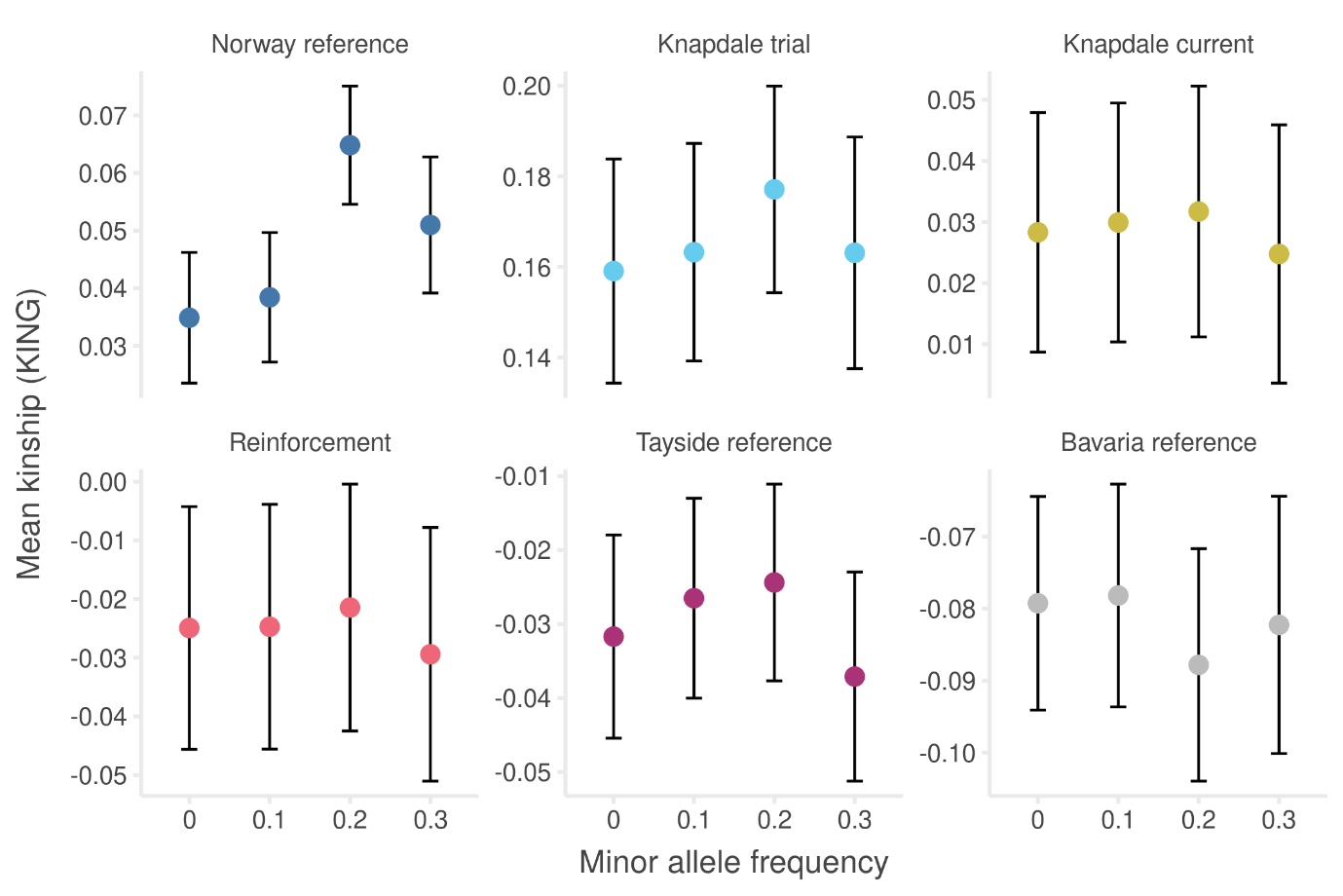
Figure S3: Varying the MAF threshold for kinship analyses using KING-robust has no significant effect on kinship estimates in any of the sample sets.


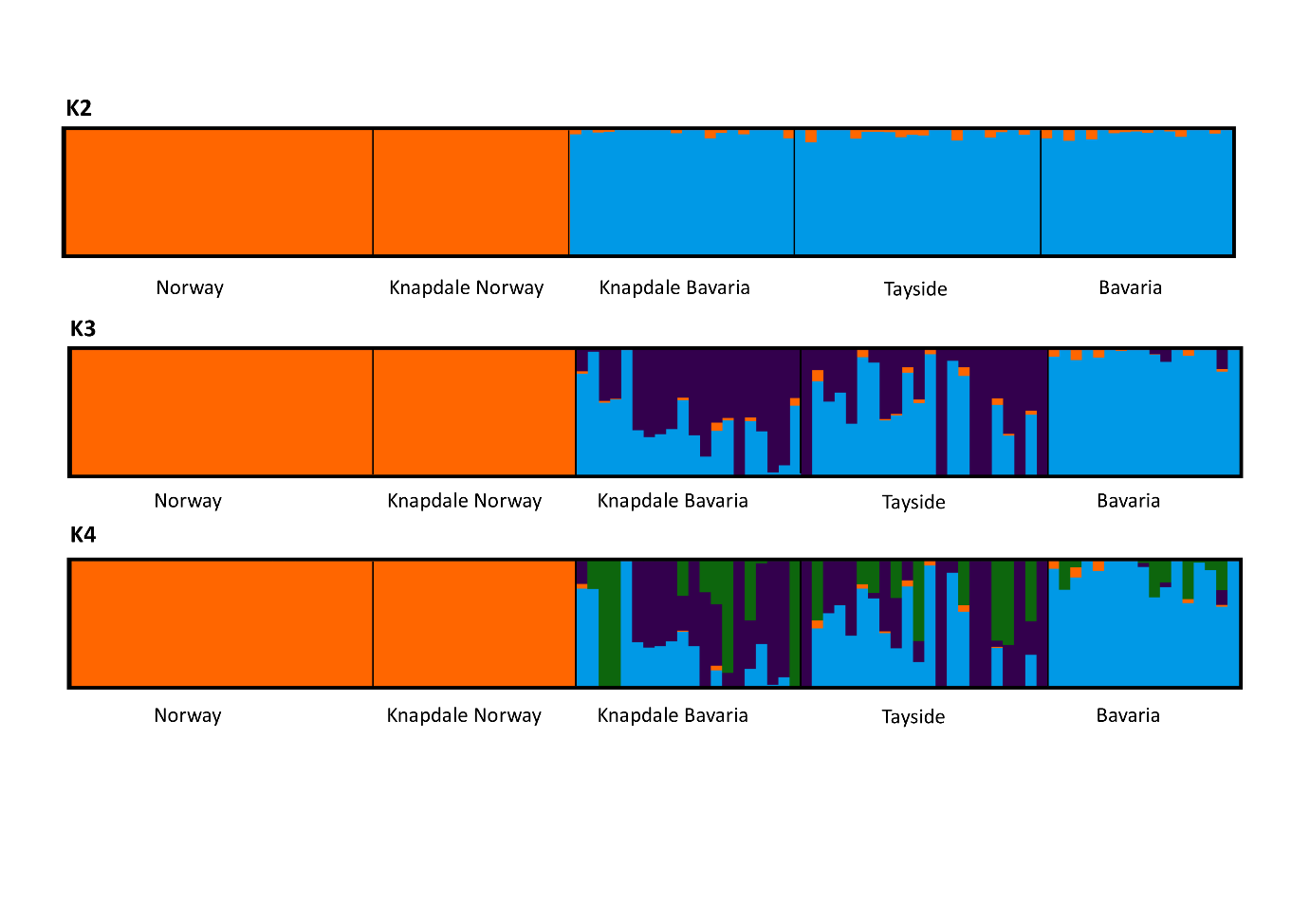


Figure S4: Population structure in Scotland’s beavers and alignment to respective source populations based on 104 individuals genotyped at 2,031 SNPs called using the American beaver reference genome and run in ADMIXTURE showing outputs for K=2 - K=4.


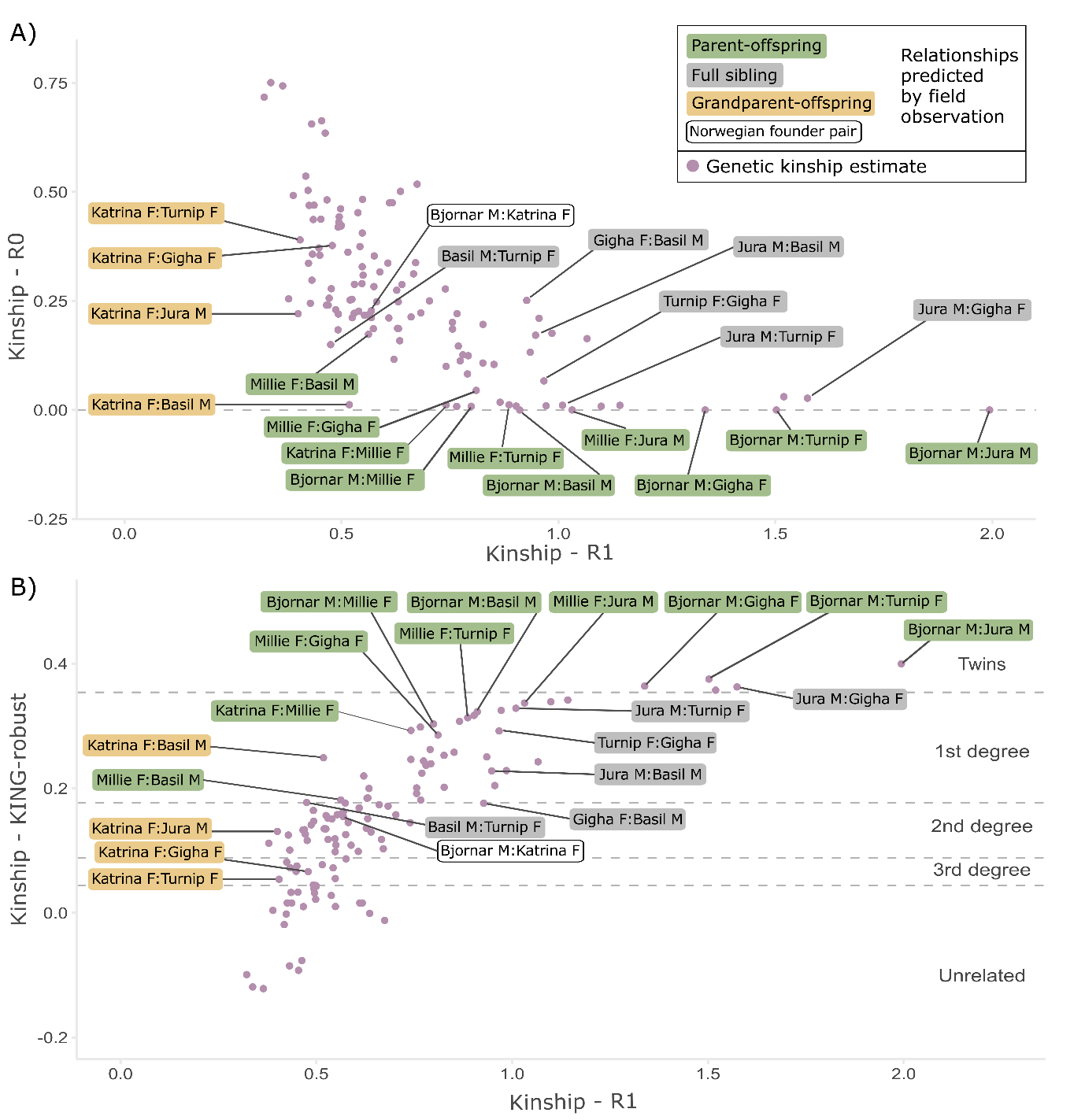


Figure S5: Relatedness within the Knapdale population in detail based on 16 individuals genotyped at 2,031 SNPs, visualised by comparison of: A) Comparison of R1 with R0. Dashed horizontal line indicates lower limit of 0 for R0; pairwise estimates of R0 = 0 theoretically correspond to a Parent-offspring relationship (but can represent the extremes of the full-sibling distribution) in a large, outbred population. Pairwise comparisons within the inbred family are labelled, where label colour indicates predicted relationships based on observational data and M=male, F=female (key top right). B) Comparison of R1 with KING-robust. Dashed horizontal lines indicate theoretical limits for familial relationships (indicated by text on right) for KING-robust kinships, based on large, outbred populations. Pairwise comparisons within the inbred family are labelled, where label colour indicates predicted relationships based on observational data (key top left).

**References**

Galla, S.J. et al. 2019. Reference genomes from distantly related species can be used for discovery of single nucleotide polymorphisms to inform conservation management. *Genes* 10(1), p. 9. Available at: www.mdpi.com/journal/genes [Accessed: 7 August 2020].

Paris, J.R., Stevens, J.R. and Catchen, J.M. 2017. Lost in parameter space: a road map for stacks. Johnston, S. ed. *Methods in Ecology and Evolution* 8(10), pp. 1360–1373. Available at: http://doi.wiley.com/10.1111/2041-210X.12775 [Accessed: 11 February 2019].

Rochette, N.C. and Catchen, J.M. 2017. Deriving genotypes from RAD-seq short-read data using Stacks. *Nature Protocols* 12(12), pp. 2640–2659. Available at: http://www.nature.com/doifinder/10.1038/nprot.2017.123 [Accessed: 11 February 2019].

Samaha, G., Wade, C.M., Mazrier, H., Grueber, C.E. and Haase, B. 2021. Exploiting genomic synteny in Felidae: cross-species genome alignments and SNV discovery can aid conservation management. *BMC Genomics 2021 22:1* 22(1), pp. 1–13. Available at: https://bmcgenomics.biomedcentral.com/articles/10.1186/s12864-021-07899-2 [Accessed: 6 October 2022].
