# Supplementary material for: The genetic legacy of the first successful reintroduction of a mammal to Britain: Founder events and attempted genetic rescue in Scotland's beaver population
Source: Evol Appl. 2023 Dec 28;17(2):e13629. doi: 10.1111/eva.13629 (PMC10853653; doi:10.1111/eva.13629)
Supplement: Supplementary file 2 — File S2 [file EVA-17-e13629-s001.docx]

**Supplementary Information 2: reference-called SNP quality control statistics**

These measures assess the quality of the final reference-based set of 2,031 SNPs genotyped in 104 individual beavers. Note that where measures are shown per population, some individuals are present in the dataset twice (see methods).

## Coverage

The mean coverage per SNP per individual was 474x, ranging from 129x to 1375x.


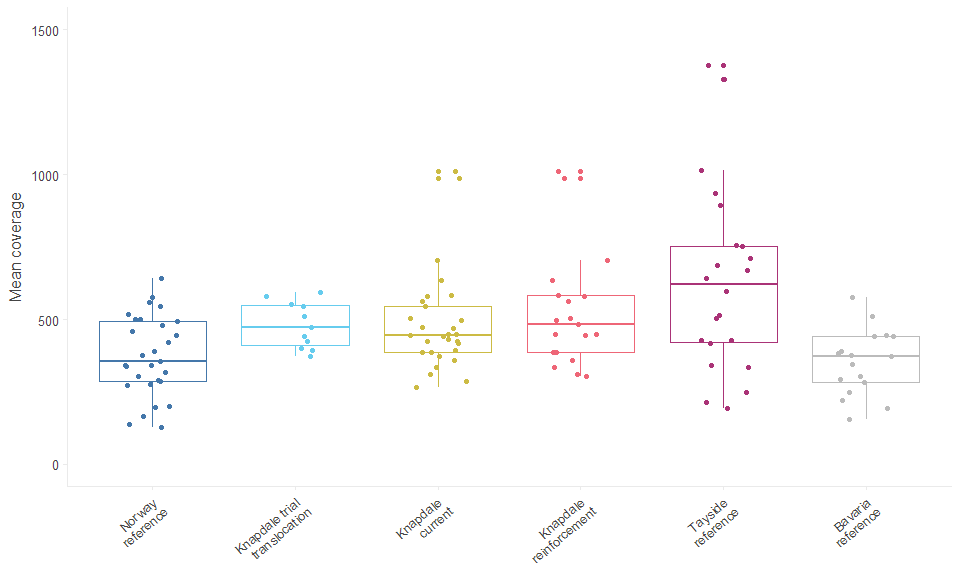


Mean coverage per SNP for each individual within each population (points). Boxplots summarise the median and interquartile ranges for each population.

## Individual genotyping rates

The mean genotyping rate per individual was 0.989, ranging from 0.903 to 1.


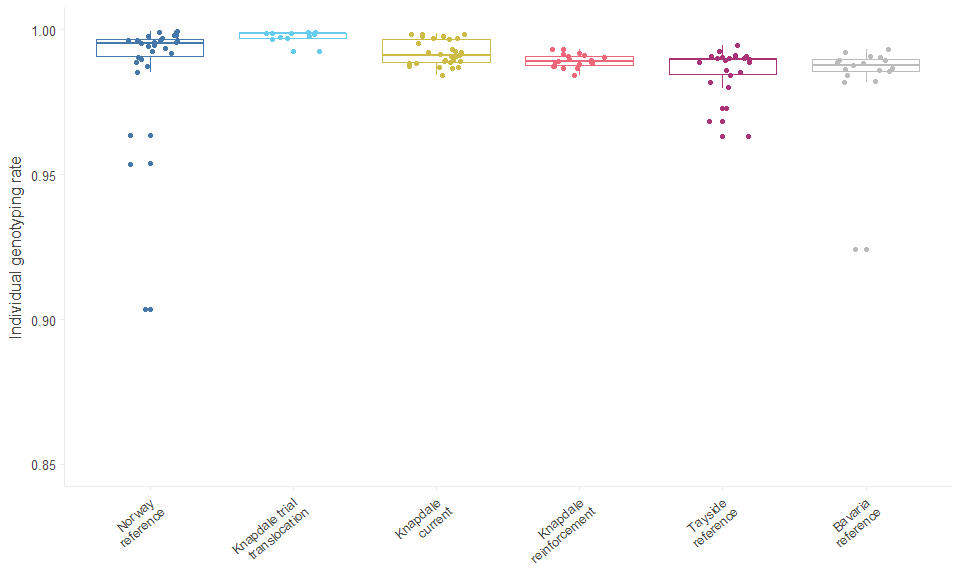


Genotyping rates for each individual within each population (points). Boxplots summarise the median and interquartile ranges for each population.

## SNP minor allele frequency (MAF)


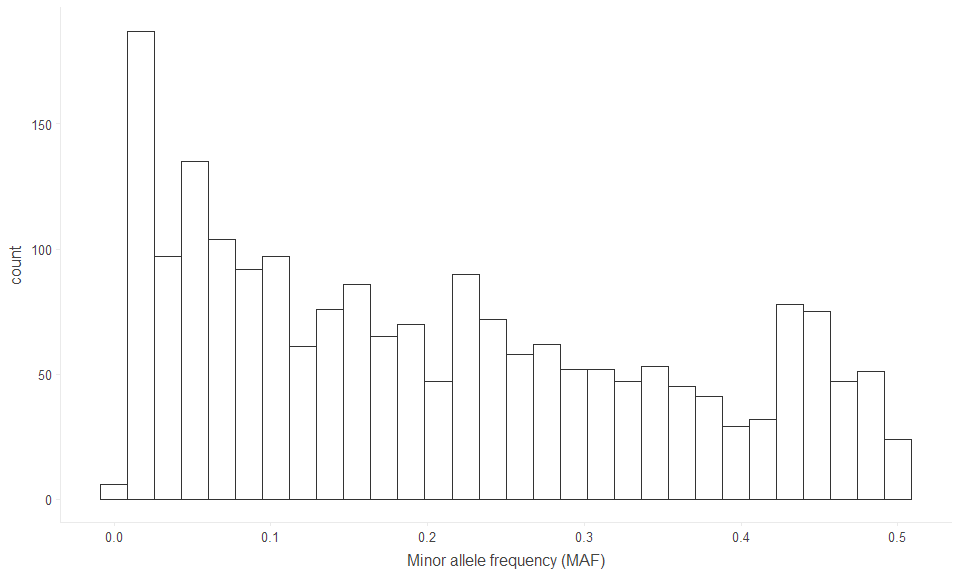


Distribution of minor allele frequencies across the 2,031 SNPs.

## SNP genotyping rate


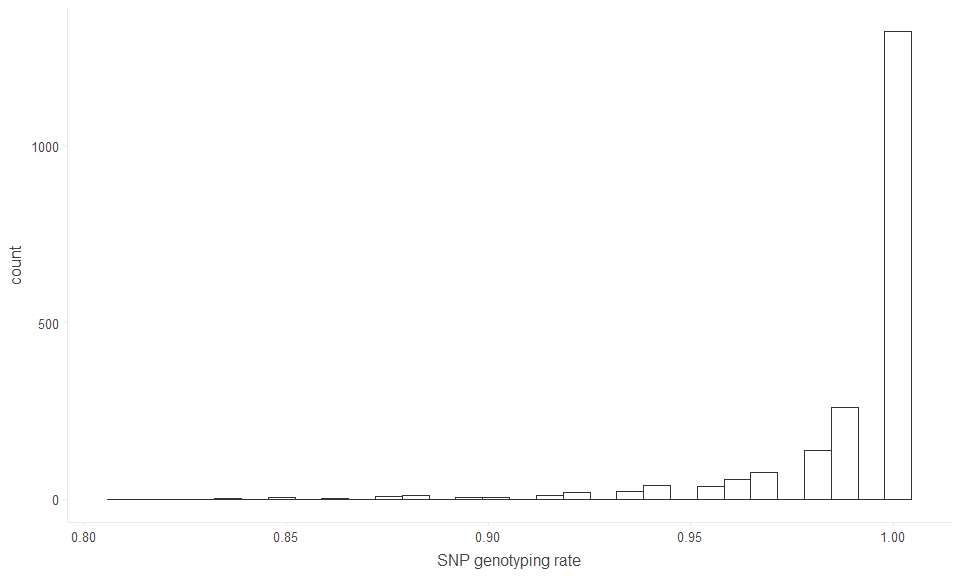


Genotyping rates across the, 2031 SNPs.
